# Flash Assembloids: A Rapid Biofabrication of a Platform for Modeling Early Glioblastoma Invasion at the Glioblastoma–Brain Organoid Interfaces

**DOI:** 10.1002/adhm.202504842

**Published:** 2026-05-11

**Authors:** Chao Liang, Henry Robert Howard, Shihui Chen, Won‐Young Choi, Summer Cao, Arthur Chien, Kevin Zou, Michael Kassiou, Fabien Delerue, Ho Sang Jung, Yeonju Park, Young Mee Jung, Yong‐Dae Kwon, Karrie M. Kiang, Gilberto Ka‐Kit Leung, Ryohichi Sugimura, Ann‐Na Cho, Sang Jin Lee

**Affiliations:** ^1^ Biofunctional Materials Division of Applied Oral Sciences and Community Dental Care Faculty of Dentistry The University of Hong Kong Hong Kong SAR China; ^2^ School of Biomedical Engineering Faculty of Engineering The University of Sydney Darlington New South Wales Australia; ^3^ The University of Sydney Nano Institute (Sydney Nano) The University of Sydney Camperdown New South Wales Australia; ^4^ Brain and Mind Centre The University of Sydney Camperdown New South Wales Australia; ^5^ Centre for Drug Discovery and Innovation The University of Sydney Camperdown New South Wales Australia; ^6^ Department of Pathology University of Tennessee Health Science Center Memphis Tennessee USA; ^7^ The Microscopy Unit Faculty of Science and Engineering Macquarie University Sydney New South Wales Australia; ^8^ School of Chemistry The University of Sydney Sydney New South Wales Australia; ^9^ Department of Genetics The University of Texas MD Anderson Cancer Center Houston Texas USA; ^10^ School of Biomedical Engineering Korea University Seoul Republic of Korea; ^11^ Department of Chemistry Institute for Molecular Science and Fusion Technology and Kangwon Radiation Convergence Research Support Center Kangwon National University Chuncheon Republic of Korea; ^12^ Department of Oral & Maxillofacial Surgery College of Dentistry Kyung Hee University Seoul Republic of Korea; ^13^ Department of Surgery School of Clinical Medicine LKS Faculty of Medicine The University of Hong Kong Hong Kong SAR China; ^14^ School of Biomedical Sciences Li Ka Shing Faculty of Medicine The University of Hong Kong Pokfulam Hong Kong SAR People's Republic of China; ^15^ College of Dentistry Kyung Hee University 26 Kyungheedae‐ro Dongdaemun‐gu Seoul Republic of Korea

**Keywords:** glioblastoma, invasiveness, tumor‐host interactions, tumor extracellular matrix, organoid fabrication

## Abstract

Glioblastoma (GBM) remains one of the most aggressive brain malignancies, characterized by rapid infiltration, therapeutic resistance, and dismal prognosis. Modeling GBM invasion in physiologically relevant systems has been hindered by the lack of reproducible platforms. Here, we present a bioengineered assembloid (ASM) system that integrates GBM cells encapsulated in self‐degradable 5% oxidized alginate microgel (5OA) with dorsal forebrain organoids (DOs) to recapitulate early tumor‐host interactions during glioblastoma invasion toward the brain. Live‐cell imaging revealed GBM self‐aggregation, leading to increased recruitment and invasion at the DO boundary, accompanied by strong cell‐cell adhesion, nuclear compaction, and the infiltration fronts enriched in SOX2^+^/Vimentin^+^ tumor populations. Transcriptomic profiling demonstrated upregulation of adhesion, integrin clustering, and mechanosensing‐associated genes, alongside downregulation of neuronal differentiation pathways, indicating a dual invasion and host suppression strategy. Comparative analyses of GBM‐only constructs and DO‐GBM ASMs revealed elevated expression of laminin subunits and enrichment of invasion‐associated pathways, including PI3K‐AKT‐mTOR and TGF‐β signaling, reflecting a shift toward an invasive state at the transcriptomic level. In vivo implantation of ASMs confirmed aggressive GBM infiltration and niche remodeling, highlighting the translational relevance. These findings suggest that it recapitulates the structural, molecular, and functional hallmarks of GBM invasion and tumor‐driven remodeling of the host brain microenvironment.

## Graphical abstract

This study presents a bioengineered assembloid (ASM) system combining glioblastoma (GBM) cells in oxidized alginate (OA) microgels with dorsal organoids (DOs). This model simulates brain tumor‐host interactions, revealing enhanced GBM invasion, altered gene expression, and aggressive infiltration patterns, demonstrating ASM as a valuable platform for studying glioblastoma dynamics.

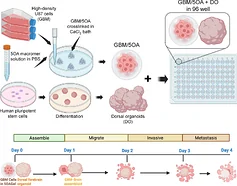

## 1 Introduction

Glioblastoma (GBM), classified by the World Health Organization as a grade IV glioma, ranks among the most aggressive and lethal primary brain tumors [1]. It is characterized by rapid proliferation, extensive invasiveness, and diffuse infiltration into surrounding brain parenchyma, which collectively impede complete surgical resection and contribute to high recurrence rates. Despite maximal surgical resection followed by radiotherapy and temozolomide‐based chemotherapy, the median survival of GBM patients remains under 15 months [2, 3, 4, 5]. Early detection, timely intervention, and precision medicine targeting tumor invasiveness are therefore critical, as they broaden therapeutic options, including surgery, radiation, and chemotherapy, while improving overall survival rate and clinical outcomes [6, 7].

Current research efforts focus on developing preclinical models that more accurately capture the complexity of GBM biology. However, traditional two‐dimensional (2D) monolayer cultures and even three‐dimensional (3D) models fail to recapitulate the dynamic tumor‐–host interactions that govern GBM progression and therapeutic resistance. Recent advances have incorporated biomaterials including hydrogels [8] and human brain organoids to model GBM behaviors in vitro [9]. For example, Chadwick et al. introduced four‐dimensional culture arrays to evaluate drug responses across patient‐derived GBM organoid‐like models, while Prior et al. demonstrated that co‐culturing dorsal cortical organoids with tumor spheroids facilitated hybrid tissue formation and altered signaling cascades [10, 11]. Despite such advances, in vitro models often rely on the artificial assembly of pre‐formed structures, which restricts the ability to model natural invasion dynamics and asymmetric sizes. Moreover, cellular aggregation in these systems typically requires several weeks to months to form assembly, limiting their utility for studying early invasion processes and dynamic tissue integration in a time‐efficient manner. As such, GBM in vitro modeling remains a need for more physiologically relevant platforms capable of capturing both the rapid onset of GBM infiltration and the dynamic transcriptional pathway of tumor‐neural interactions.

**FIGURE 1:**
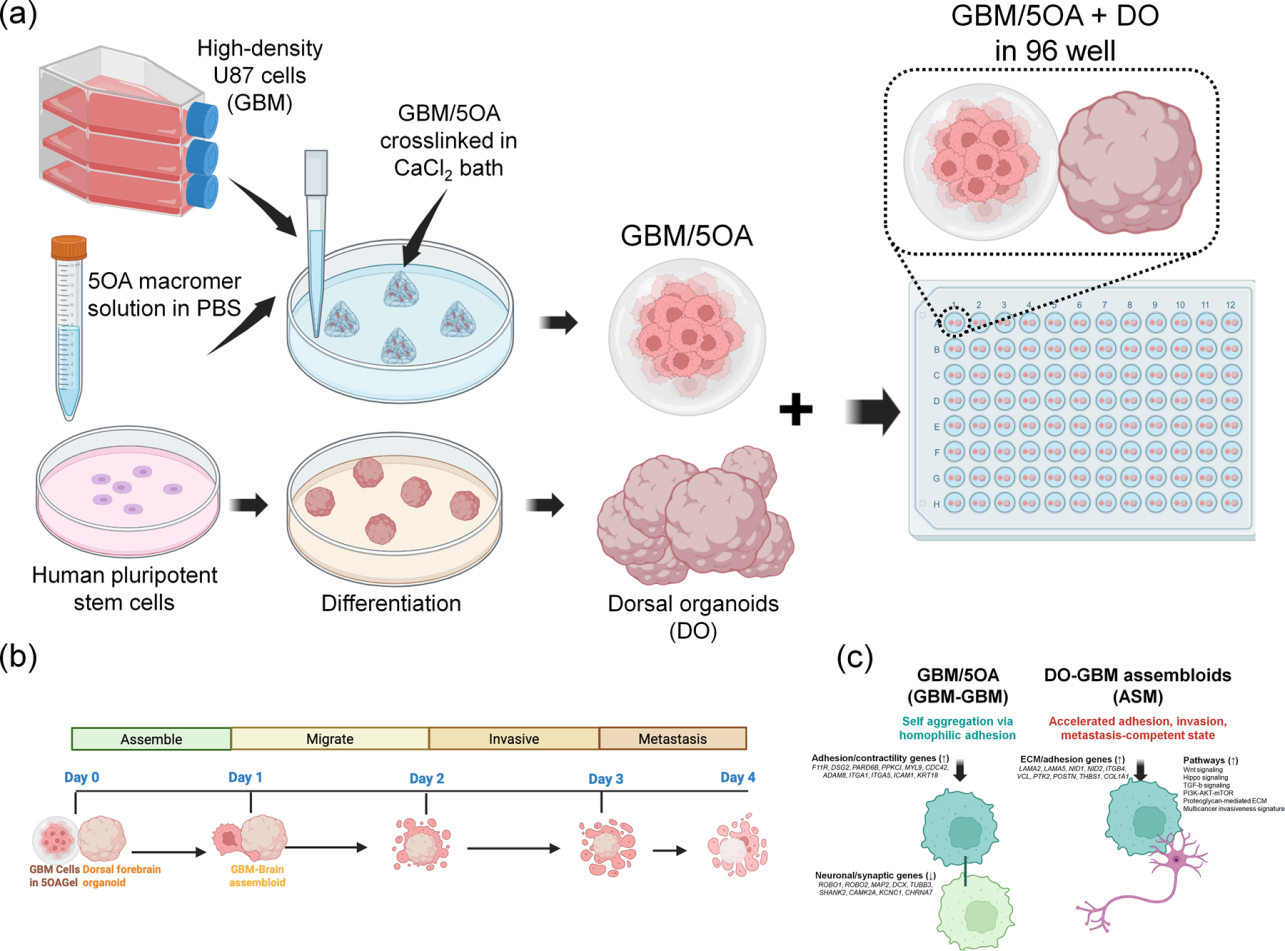
Schematic illustration of in vitro DO‐GBM ASM model. (a) Generation of an ASM model using a co‐culture method using preformed DOs with GBM/5OA microgels and further incubated in a plate to form self‐assembling DO‐GBM ASMs. (b) Schematic figure of the time‐course progression of distinct stages of ASMs, following encapsulation of GBM cells within 5% OA microgels (GBM/5OA) and co‐culture with DOs. (c) Molecular pathway of GBM spheroids (GBM‐GBM) versus DO‐GBM ASMs. GBM spheroids predominantly exhibit self‐aggregation via homophilic adhesion, supported by upregulation of adhesion and contractility‐associated genes (*F11R, DSG2, ITGA1/5, MYL9, CDC42, ICAM1*). By contrast, DO‐GBM ASMs show laminin‐integrin clustering (*LAMB1, ITGB1, ITGA6, TLN1, VCL*) and activation of oncogenic signaling cascades, including PI3K‐AKT‐mTOR and TGF‐β, alongside enrichment of invasion‐associated signatures (ANASTASSIOU_MULTICANCER_INVASIVENESS_SIGNATURE). Created using Biorender under a license.

To address this gap, in this study, we engineered an oxidized alginate (OA) microgel platform that enables near‐instant formation (within 1 s) of high‐density GBM aggregates in large yield (∼60 organoid formation per minute), which can subsequently fuse with mature dorsal forebrain organoids (DOs) to form asymmetric assembloids (ASMs). In our recent work, Lee et al. reported that OA microgels undergo rapid self‐degradation in culture, enabling encapsulated cells to spontaneously aggregate without the need for enzymatic degradation or chelation [12]. OA microgels are commonly synthesized by treating alginate with sodium periodate (NaIO_4_) to introduce dialdehyde groups [13, 14], which confer their self‐degrading properties suitable for tissue integration for both in vitro and in vivo environments [14]. Leveraging this feature, we first encapsulated GBM cells in OA microgels, followed by co‐culturing with DOs (Figure 1a) to fabricate heterogeneous ASM structures, which started to form within 1 day. This platform preserves tissue‐level integrity while enabling real‐time modeling of tumor migration and invasion (Figure 1b,c).

Our OA microgel approach offers distinct advantages over the direct co‐culture of dissociated cells as well as symmetric organoid assembloid. First, it establishes a controlled initial interface by generating a defined, 3D “tumor nodule” that more closely resembles an early‐stage glioblastoma focus within a confined micro‐niche, rather than relying on randomly dispersed single cells [15]. Second, it enables replication of ECM remodeling by the self‐degradation of the OA matrix which initially models the highly stiff mechano‐physiological environment of GBM cells condensation during early tumor cell migration [16], while its subsequent degradation recapitulates a key feature of tumor progression, namely local matrix breakdown that facilitates invasion [17]. Third, this strategy enhances physiological relevance compared with conventional ASM methodologies, which often combine brain organoids and tumor components of comparable size and cell numbers; in vivo, however, GBM frequently emerges as a relatively small lesion, ranging from single cells to compact microclusters, which subsequently infiltrate a much larger brain tissue environment to form colonies [18, 19, 20, 21, 22]. By establishing a size‐asymmetric configuration, which subsequently infiltrates as a small cluster a much larger brain tissue environment and then self‐aggregates and proliferates inside [23, 24, 25], therefore our model better captures this native spatial relationship and enables a more realistic assessment of early invasion and infiltration dynamics [26]. Fourth, the aggregate‐organoid configuration facilitates a clearer framework for interrogating tumor‐brain interaction in cellular and tissue‐level, including the pronounced tropism of glioblastoma toward neural tissue. Furthermore, this well‐established microgel‐based aggregate approach improves experimental reproducibility compared with patient‐derived explants‐based organoids by standardizing the initial tumor cell density and spatial positioning relative to the brain organoid, thereby reducing variability in the onset of interactions compared to the addition of dissociated cells. Finally, compared to the ex vivo model directly using GBM ex vivo sample for GBM organoid generation, which contains high heterogeneous identity, our model shows stable and standardized brain organoid formation and their interaction with glioblastoma cells consistently in the laboratory setting but also be utilized in a clinical studies by using our identified pipeline to evaluate the early tumor‐brain interaction and invasion in spatiotemporal resolution [27, 28, 29].

To demonstrate it, herein, we characterized this platform to demonstrate that ASMs not only enabled rapid and reproducible modeling of tumor‐host interactions but also recapitulate key hallmarks of GBM biology during early stages of interaction. By integrating live imaging, histology, immunofluorescence, and transcriptomic profiling via RNA sequencing (RNA‐seq), we revealed time‐resolved insights into tumor aggregation, invasion, and metabolic adaptation. Furthermore, through an in vivo implantation study, we established its translational relevance as a preclinical tool for studying GBM pathogenesis and for accelerating the development of patient‐derived tumor models.

## 2 Results and Discussion

**FIGURE 2:**
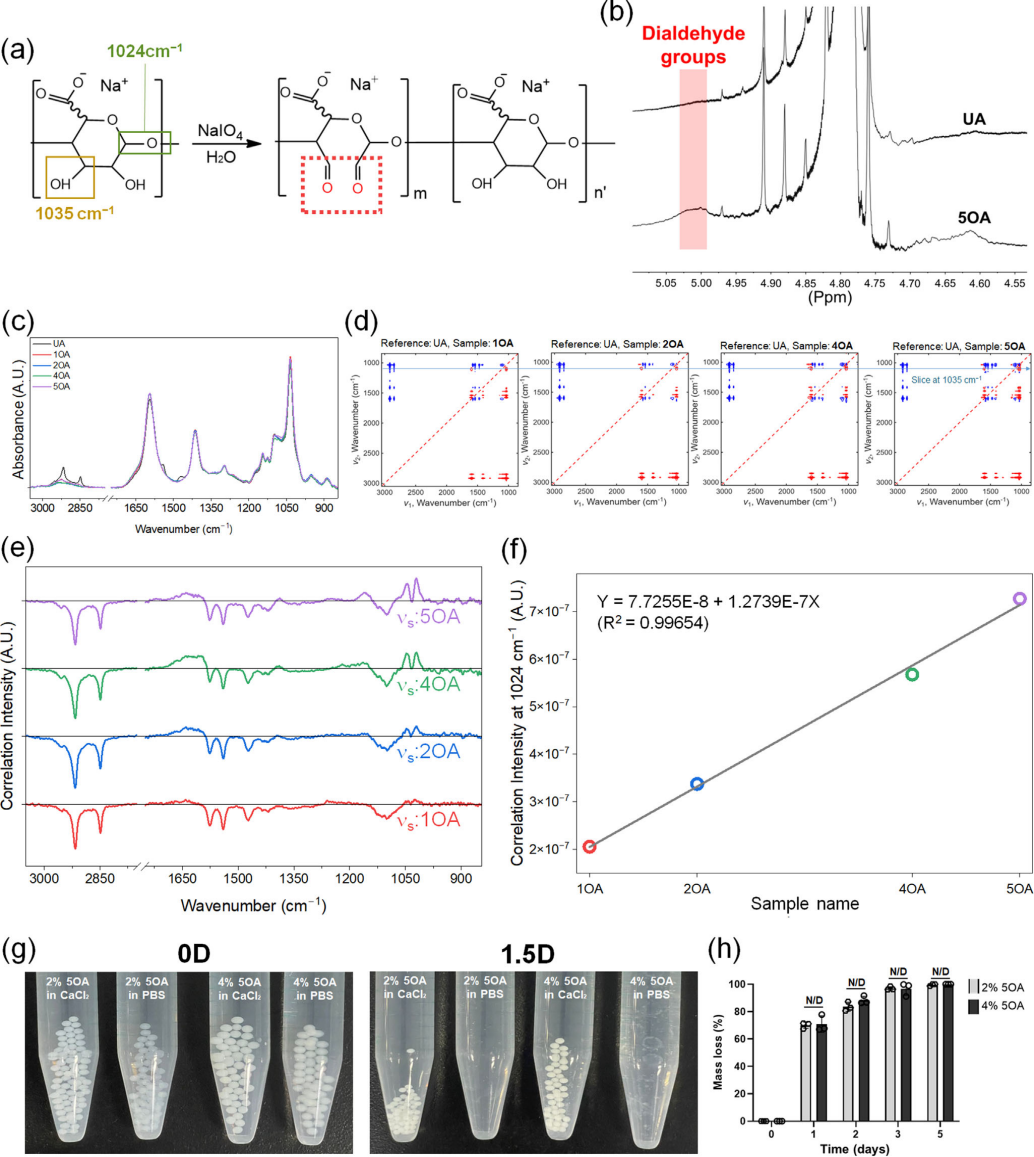
Preparation of OA and characterization of its oxidation degree and degradation behavior. (a) Schematic illustration of alginate oxidation, including band assignments for the C‐O‐C stretching vibration of glycosidic linkage (1024 cm^−1^) and the C‐O stretching vibration of secondary alcohol group (1035 cm^−1^). (b) 1H‐NMR spectra of unmodified UA and 5OA showing the appearance of signals corresponding to newly formed dialdehyde groups in 5OA. (c) IR spectra of UA and OA samples with increasing theoretical oxidation degree (1OA, 2OA, 4OA, and 5OA). OA gels were prepared as 5 wt.% concentration to dissolve DI water and stored at room temperature for 24 h before analysis. (d) Asynchronous 2T2D correlation spectra, derived from cross‐product equation: Ψ(*ν*_1_, *ν*_2_) = 1/2[*s*(*ν*_1_)*r*(*ν*_2_) − *r*(*ν*_2_)*s*(*ν*_1_)]. (e) Slice spectra at 1035 cm^−1^ extracted from 2T2D correlation spectra (d), and (f) quantitative determination of oxidation degree based on the linear calibration plot (*R* ^2^ = 0.99654) between the oxidation degree and correlation intensity at 1024 cm^−1^. (g) The gross view of 2% and 4% 5OA microgels incubated in CaCl_2_ or PBS at room temperature validating degradation kinetics (*n* = 75). Both formulations underwent completely degradation in PBS, whereas structural integrity was maintained in CaCl_2_. (h) Quantitative analysis of degradation kinetics showing that 2% and 4% 5OA microgels were almost completely degraded within 3 days (*n* = 3).

In this study, OA was synthesized via NaIO_4_ for the oxidation process (Figure 2a, red square) [30]. The newly introduced dialdehyde groups were confirmed by the ^1^H‐NMR spectrum of OA, which revealed a new proton peak around 5 ppm compared to unmodified alginate (Figure 2b, red bar). This signal is attributed to the protons of hemiacetal structures formed upon the reaction of the newly generated aldehyde groups with adjacent hydroxyl groups, indirectly confirming successful oxidation [31].

To confirm the oxidation degree in OA, the IR spectra of UA and various OA samples (1OA, 2OA, 4OA, and 5OA) were compared (Figure 2c–f). No clear correlation was observed between spectral intensity changes and the degree of oxidation. In particular, the C═O stretching band near 1720 cm^−1^ and the broad O─H stretching band near 3500cm^−1^, which are the primary indicators of the conversion of C─OH groups into C═O groups, were difficult to discern (Figure 2c). This limited sensitivity arises from two main factors: (i) the concentration of newly formed C═O groups is likely too low to be detected by a conventional IR spectrum near 1720 cm^−1^, and (ii) the O─H region near 3500 cm^−1^cm is obscured by the baseline interference caused by the H_2_O spectrum subtraction.

To overcome these detection limits and enable quantitative analysis of the oxidation degree, we applied two‐trace 2D correlation spectroscopy (2T2D‐COS) using UA as the reference spectrum (*r*) and OA as the sample spectrum (*s*) (Figure 2d). Following the quantitative approach suggested by Jin et al. [32], slice spectra at 1035 cm^−1^ were extracted from the asynchronous 2T2D correlation spectra. Even with the application of 2T2D‐COS, the C═O stretching band near 1720 cm^−1^ degree is not easily distinguishable. This selection is based on the observation that, during oxidation, the intensity of the band at 1035 cm^−1^ assigned to C─O stretching of secondary alcohols in the alginate ring [33] decreases as the C─OH groups are consumed. In contrast, the intensity of the band at 1024 cm^−1^ assigned to C─O─C stretching of the glycosidic linkages [34] remains relatively constant in intensity. To quantitatively evaluate these changes, we employed the asynchronous correlation intensity calculated from the cross‐product of the spectral intensity vectors as defined in 2T2D‐COS theory: Ψ(*ν*_1024_, *ν*_1035_) = 1/2 [*s*(*ν*_1024_)*r*(*ν*_1035_) − *r*(*ν*_1024_)*s*(*ν*_1035_)] (Figure 2e,f). By plotting this correlation intensity at 1024 cm^−1^ against the series of oxidation samples, we established a calibration model that exhibited a strong linear relationship (*R* ^2 ^= 0.99654) (Figure 2f). Based on this model, the degrees of oxidation for the 1OA, 2OA, 4OA, and 5OA samples were determined to be 1.006%, 2.042%, 3.850%, and 5.102%, respectively. These results demonstrate that the fingerprint region, when analyzed using 2T2D‐COS, can serve as a highly sensitive probe for quantifying chemical transformations that are not detectable in the primary functional group regions. Based on the results, we selected the 5OA condition and continuously applied it to highly oxidized OA microgels to promote rapid degradation. The higher degree of oxidation accelerated OA breakdown, enabling the formation of 3D tissue organization [14].

In degradation tests, conditioned microgels fabricated from 2% and 4% 5OA were monitored over 1.5 days in calcium chloride (CaCl_2_) and phosphate‐buffered saline (PBS), respectively. The 2% or 4% 5OA microgels presented a uniformly spherical structure in CaCl_2_ and PBS conditions on day 0 (Figure 2g). On day 1.5, most 2% or 4% 5OA microgel structures persisted in CaCl_2_ conditions; on the other hand, most 2% or 4% microgels had disappeared in PBS conditions. As shown by the quantitative measurements in Figure 2h, most 2% and 4% 5OA microgels degraded within 1–2 days, which matched the gross appearance observed in Figure 2g. The actual degradation of 5OA microgels in physiological conditions is attributed to a combination of hydrolysis and alkali‐catalyzed β‐elimination of the dialdehyde‐modified guluronate residues. Under physiological conditions (pH 7.4, 37°C), β‐elimination is considered the predominant pathway, and this process is accelerated by the open‐chain conformation introduced by periodate oxidation [35]. Both mechanisms contribute to the controlled degradation kinetics observed in our system [14, 36]. The degradation products of OA are small, non‐cytotoxic oligosaccharides that are readily cleared from the system and are not expected to interfere with cellular behavior in our ASM model [14, 30, 37].

The DOs were matured before the experiment as validated by immunocytochemical staining ofcellular populations and mature matrkers in their spatial orientation (Figure S1). DOs exhibited PAX6 as indicator of the dorsal forebrain progenitor,  radial glial cell and neuroectoderm identity [38, 39, 40]. Additionally, our DOs showed widespread FOXG1 expression confirmed forebrain regional specification. Robust neuronal differentiation was demonstrated by TUJ1 (βIII‐tubulin) and MAP2 staining, indicating the presence of developing neuronal populations and organized neural tube structures. In addition, the coexistence of neural progenitor and proliferative populations was evidenced by Nestin and Ki67 staining, respectively. Collectively, these findings confirm that the generated organoids possess dorsal forebrain identity and contain both progenitor and mature neuronal populations consistent with established dorsal forebrain organoid models [39].

**FIGURE 3:**
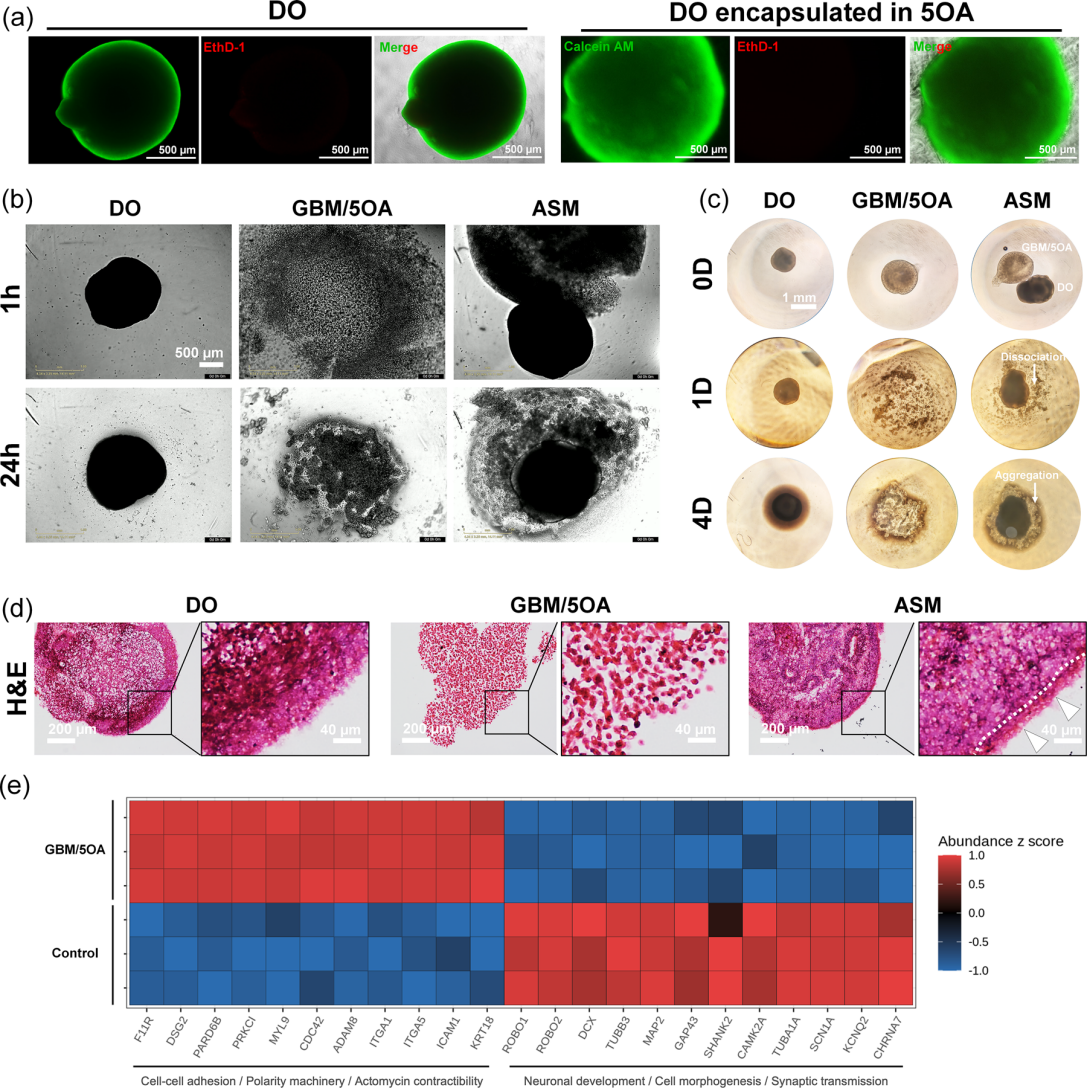
Generation of DO, GBM/5OA, and ASM and characterization of tissue integrity. (a) Live/Dead viability assessment of DOs cultured alone and following encapsulation in 4% 5OA microgels. (b) Live‐cell monitoring of DO, GBM, and ASM. The GBM group shows GBM aggregation internally and ASM formation between DO and GBM, indicating the assembly of GBM around the DO. Time‐lapse imaging showed degradation of GBM microgels and subsequent GBM cell aggregation at 1 day. (c) After 4 days of co‐culture, GBM cells were released from the microgels and aggregated around the DO. In the ASM group, a condensed structure formed via DO‐GBM assembly. Accelerated GBM recruitment to the DO surface was observed by day 4, with evidence of directed migration and peripheral compaction. (d) DOs, self‐assembling GBMs, and ASMs were analyzed using H&E staining at 2 days post‐culture. GBM cells strongly adhered to the DO (white arrow), indicated by translucent eosinophilic cytoplasm adjacent to the DO nuclei. (e) Heat map showing gene expression profiles in reference fetal brain organoids and GBM/5OA aggregates. The map includes genes related to aggregation and neurodevelopment. Values represent row‐wise *z*‐scores per gene. Columns reflect three biological replicates, respectively (GBM/5OA vs. Control as human fetal dorsal brain organoids from a public dataset (GSE248479)), grouped by condition.

To evaluate the biocompatibility of the 5OA microgel, a live/dead assay was performed on DOs culture alone and DOs encapsulated within 5OA microgels (Figure 3a). Live cells were visualized with Calcein‐AM (green), whereas dead cells were labeled with EthD‐1 (red). Both conditions exhibited high viability with minimal cell death, indicating that encapsulation within OA did not induce detectable cytotoxicity under the culture conditions used. As an initial step (Figure 3b), we characterized GBM cell aggregation kinetics within 5OA microgels, where cells resided in high‐density GBM suspensions. Microscope images demonstrated that 5OA microgels underwent progressive degradation from one hour, consistent with progressive hydrolytic degradation under culture conditions which includes GBM‐secreted enzymes and oxidative media exposure [37]. Following degradation, spontaneous cellular aggregation and organization were observed by 24 h, leading to the formation of compact aggregate structures (Movie S1). This assembly is likely driven by intrinsic GBM cell‐cell adhesion and motility, behaviors previously linked to tumor self‐organization capacity and integrin‐mediated contractility. Collectively, these findings demonstrated that the 5OA platform not only supported rapid matrix remodeling but also enabled autonomous GBM spheroid formation, creating a permissive interface for subsequent co‐culture with neural organoids such as DOs.

To visualize the dynamic interactions at the tumor‐organoid interface, we conducted live‐cell time‐lapse imaging over 24 h following co‐culture of GBM/5OA microgels with mature DOs, in order to form integrated ASMs (Figure 3, Movie S2). Between 1 and 24 h post‐co‐culture, GBM‐encapsulated 5OA microgels visibly degraded, releasing individual tumor cells, which subsequently aggregated around the DOs (Figure 3b). By day 1, the degraded gel matrix permitted spontaneous GBM aggregation onto the DO surface as compact clusters (Figure 3c). Notably, we observed accelerated tumor cell migration and recruitment around the DO surface through day 1 to day 4, suggesting an early onset of tumor‐host interaction with strong neurotropic migration of glioblastoma (Figure S3). From day 0 to day 1, we observed the formation of compact GBM clusters localized at the organoid surface, indicating initial tumor adhesion. By day 2, GBM cells exhibited peripheral spreading behavior, followed by active invasion into the organoid parenchyma on day 3, after OA gel degradation enabling physical cell‐cell adhesion. By day 4, the system displayed invasive phenotypes, characterized by dense nuclear aggregation at the tumor front and deep infiltration into host tissue domains, which are hallmarks consistent with clinical features of glioblastoma progression. This spatiotemporal sequence, adhesion, peripheral migration, parenchymal invasion, and aggressive infiltration, recapitulates key stages of GBM pathogenesis and underscores the utility of this platform for modeling the progression of high‐grade glioblastoma in vitro.

Imaging analysis further demonstrated that GBM aggregation was significantly enhanced in the presence of DOs, compared to 5OA‐only GBM controls (Figure S2). This reveals that neural organoid‐derived cues likely contribute to chemoattractive or mechanotactic signaling, promoting GBM cell clustering and polarization. Histological analysis using hematoxylin and eosin (H&E) staining confirmed preserved tissue boundaries and robust cellular adhesion in ASMs, signifying successful structural integration (Figure 3d). Importantly, compared to GBM/5OA controls, ASMs exhibited preserved tissue boundaries and robust cellular adhesion, indicating successful structural cell‐cell integration (white arrow in ASM group). Furthermore, in Figure S3a, GBM cells were observed to adhere to and penetrate the outer layers of the DO, evidenced by translucent eosin‐stained cytoplasmic regions surrounding darkly stained nuclei in the DO compartment, highlighting active adherence (Figure S3b–i, white arrow) and invasive remodeling (Figure S3b‐ii, white arrow) at the tumor‐organoid interface. The stem‐like phenotype and malignancy of GBM cells were supported by Nestin immunostaining (Figure S3b), and their invasive phenotype was supported by pronounced infiltration across the interface, consistent with pathways implicated in GBM migration, including PI3K‐AKT‐mTOR signaling [41]. The histological signatures reflected active invasion and remodeling at the DO‐GBM interface, thereby validating the ASM platform for modeling early GBM adherence and infiltration.

To investigate the molecular programs underlying this rapid aggregation and tumor‐host interface formation, we performed transcriptomic profiling by integrating our RNA‐seq data with reference fetal brain organoid datasets (Figure 3e) [42]. The GBM/5OA aggregate showed significant upregulation of genes associated with cell‐cell adhesion, polarity, cytoskeletal remodeling, and contractility, including F11R, DSG2, PARD6B, PRKCI, MYL9, CDC42, ADAM8, ITGA1, ITGA5, ICAM1, and KRT18. These transcriptional profiles are consistent with activation of mesenchymal‐like programs that support both homophilic aggregation and pro‐invasive phenotype. Notably, ITGA1 and ITGA5 are critical integrins mediating adhesion to extracellular matrix (ECM) components, while CDC42 and MYL9 coordinate cytoskeletal dynamics and contractile force generation. ADAM8, a disintegrin and metalloprotease, has been implicated in ECM remodeling and immune modulation, and its upregulation supports an invasive phenotype [43, 44]. Additionally, increased ICAM1 expression suggests potential roles in tumor‐endothelial adhesion and inflammatory signaling [45, 46, 47]. In contrast, genes associated with neuronal differentiation and synaptic signaling, including *ROBO1*, *ROBO2*, *MAP2*, *DCX*, *TUBB3*, *SHANK2*, *CAMK2A*, *KCNC1*, and *CHRNA7*, were consistently downregulated. These genes are associated with neuronal maturation, axon guidance, and synaptic function, and their reduced expression indicates a transcriptional shift away from neuronal differentiation programs during GBM‐neural interaction. Importantly, this finding reflects altered gene expression states within the ASM rather than complete loss of neuronal marker expressionor neuronal structures. Downregulation of axon‐guidance receptors (ROBO1, ROBO2) may reduce inhibitory cues that normally constrain cellular migration, consistent with the acquisition of a more permissive invasive microenvironment. These observations align with reports of glioma cells co‐opting brain‐like environments while maintaining transcriptional plasticity to promote invasion [9, 22, 48, 49, 50, 51, 52, 53, 54, 55]. Taken together, these data emphasize that the GBM cells rapidly aggregate to form compact, self‐organized invasion fronts that exhibit enhanced GBM‐GBM and GBM‐host adhesion. This accelerated aggregation and affinity, evident within several hourss, represents a significant advancement over conventional GBM organoid‐brainorganoid assembly, which often require several weeks for meaningful infiltration [50, 56, 57, 58, 59]. Moreover, the observed compaction mechanics and molecular signatures point to early biomechanical and transcriptional reprogramming visible in aggressive, treatment‐resistant phenotypes.

**FIGURE 4:**
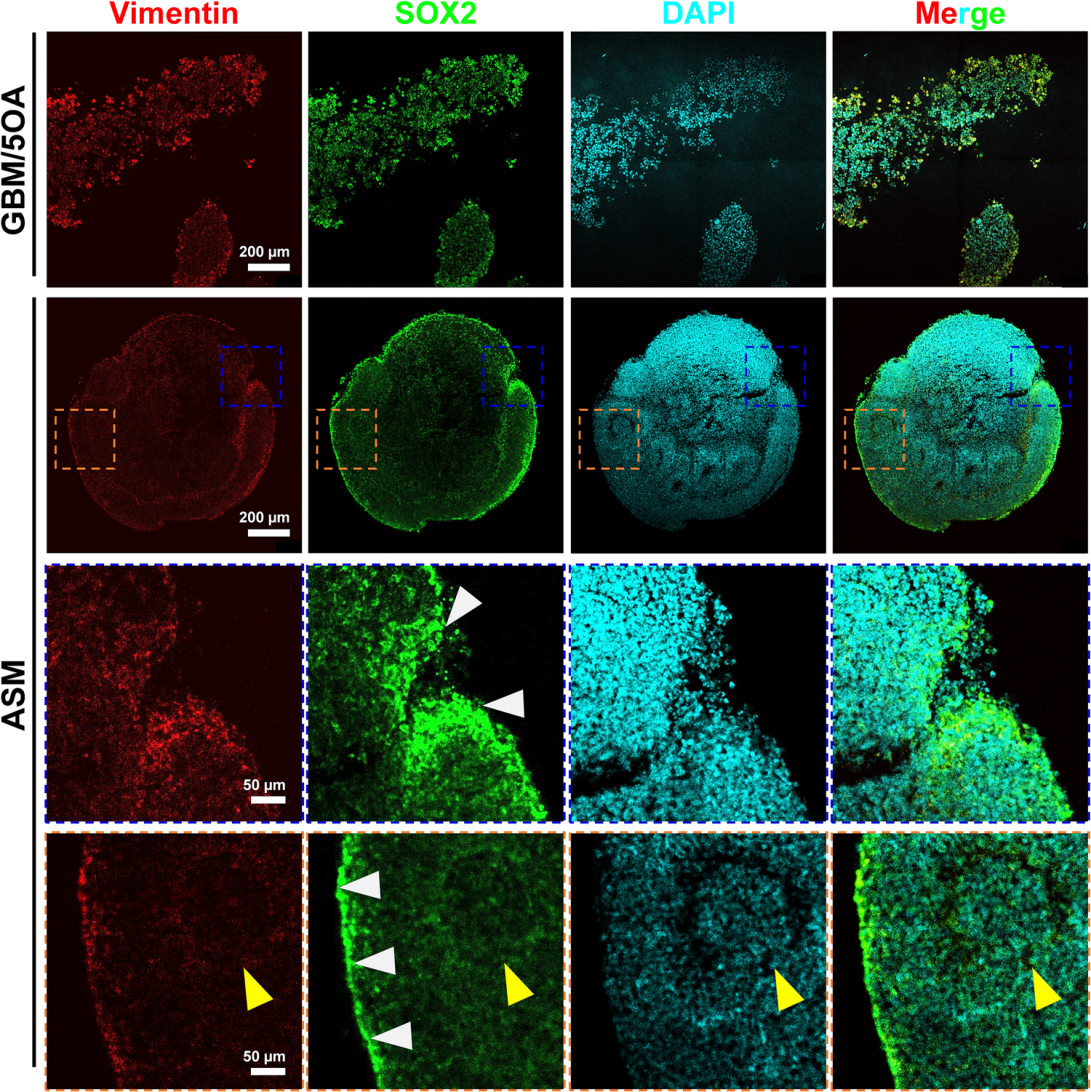
Immunofluorescence analysis of GBM and ASM. Confocal images showing Vimentin (red), SOX2 (green), and nuclear counterstaining with DAPI (blue). In GBM‐only aggregates, Vimentin expression was observed throughout the samples, with SOX2^+^ proliferative cells enriched in the majority of the population, reflecting the intrinsic self‐organization of mesenchymal and stem‐like compartments (top panel). In ASMs, GBM cells formed compact invasive fronts at the tumor‐organoid boundary, where Vimentin^+^ processes extended into the organoid and SOX2^+^ clusters co‐localized at the invasion sites (white arrowheads). Yellow arrows highlight regions of disrupted organoid cytoarchitecture, consistent with tumor‐driven remodeling of the host tissue. High‐magnification insets reveal nuclear crowding and structural compaction at the interface, supporting activation of the mechano‐transductive pathway. Merged channels illustrate the direct spatial juxtaposition of GBM and DO compartments, capturing heterotypic affinity and early tumor–neural integration.

To characterize the early tumor‐host interface and invasive behavior of GBM within our aggregate platform, we performed immunofluorescence staining on GBM and ASMs to compare cellular orientation and tissue‐level structure (Figure 4). The results revealed that GBM/5OA exhibited prominent expression of Vimentin, an immunoreactive canonical mesenchymal marker [60], and a central population of SOX2^+^ cells, consistent with a stem‐like, proliferative compartment [61]. These features suggest that the organization of GBM cells within spheroids is governed by intrinsic adhesion forces and self‐assembly dynamics, mediated by adhesion, junctions, integrin‐ECM interactions, and cytoskeletal tension [62, 63]. In the DO‐GBM co‐cultured ASMs, Vimentin^+^/SOX2^+^ GBM cells rapidly accumulated at the organoid boundary and penetrated the organoid structure, suggesting increased affinity for neural‐derived cues [48]. Their co‐localization at invasion fronts supports the idea that the neural microenvironment not only promotes mesenchymal‐driven infiltration (Vimentin^+^) but also stabilizes stem‐like tumor populations (SOX2^+^), thereby coupling invasion with self‐renewal through niche‐associated signaling pathways including NOTCH, TGF‐β, and nitric oxide (NO) [64]. These invasion zones showed intense nuclear crowding and spatial compaction, indicative of mechano‐transductive remodeling potentially involving pathways such as YAP/TAZ and NF‐κB/TGF‐β signaling [65]. This behavior mirrors clinical observations of GBM, in which tumor cells exploit progenitor‐rich niches to enhance plasticity, remodel the surrounding brain cytoarchitecture [66, 67], and drive a more aggressive pattern of tumor progression [68]. The observed Vimentin gradients at invasion fronts are consistent with EMT‐like programs and integrin‐mediated adhesion processes, while the dense nuclear clustering suggests biophysical compaction and potential activation of mechano‐transduction pathways such as YAP/TAZ [69, 70]. DAPI counterstaining further revealed zones of nuclear distortion and cytoarchitectural stress at the tumor‐host boundary— resembling so‐called “plasticity hubs”— regions where stem‐like and mesenchymal circuits intersect to promote infiltration, survival, and potential therapeutic resistance [48, 71]. This architecture aligns with the concept of relapse‐initiating reservoirs in vivo, where glioblastoma cells exploit progenitor‐rich or perivascular niches to survive therapy and reseed growth [27, 72, 73].

Importantly, such features emerged within several hourss of co‐culture (Figure 3b,c), a markedly accelerated timeline compared to conventional organoid‐organoid fusion assays that often require 2–4 weeks before cell infiltration and tissue scale reconstruction [74, 75]. This rapid onset highlights not only the intrinsic affinity of GBM cells for neural substrates, but also the capacity of early neural niches to act as invasion‐permissive compartments. Recent work shows that patient‐derived GBM spheroids invade soft, brain‐like matrices through hyaluronic acid (HA)‐CD44‐ezrin coupling, displaying a biphasic sensitivity to HA concentration [76]. Such interactions suggest that even subtle ECM variations in developing tissue can prime tumor cells for motility. Consistent with this, organoid infiltration assays reveal that GBM spheroids, unlike neural progenitor spheroids, spontaneously integrate into early cerebral organoids within 48 h, showcasing how developmental microenvironments may also accelerate invasive transitions. Mechanistically, the nuclear deformation and spatial crowding observed in these models are consistent with activation of mechanosensitive pathways, which transduce physical stress into transcriptional programs that drive invasion and therapeutic resistance. Clinically, this resonates with the tendency of recurrent tumors to re‐emerge along perivascular and neurogenic niches, where glioma stem‐like cells exploit supportive microenvironments to sustain proliferation and invasion despite therapy [40, 77, 78]. These insights extend beyond morphology, offering a window into how biophysical and molecular cues coalesce to establish invasion‐competent micro‐domains. While these findings highlight the translational potential of DO‐GBM ASMs, validation will require live single‐cell imaging, lineage tracing, and systematic testing across diverse patient lines to confirm whether such accelerated adhesion universally predicts aggressive, relapse‐initiating phenotypes. Moreover, integrating immune or vascular compartments into this platform could reveal whether the same niches that promote GBM plasticity also condition immune evasion or angiogenic mimicry, broadening its utility as a preclinical tool.

**FIGURE 5:**
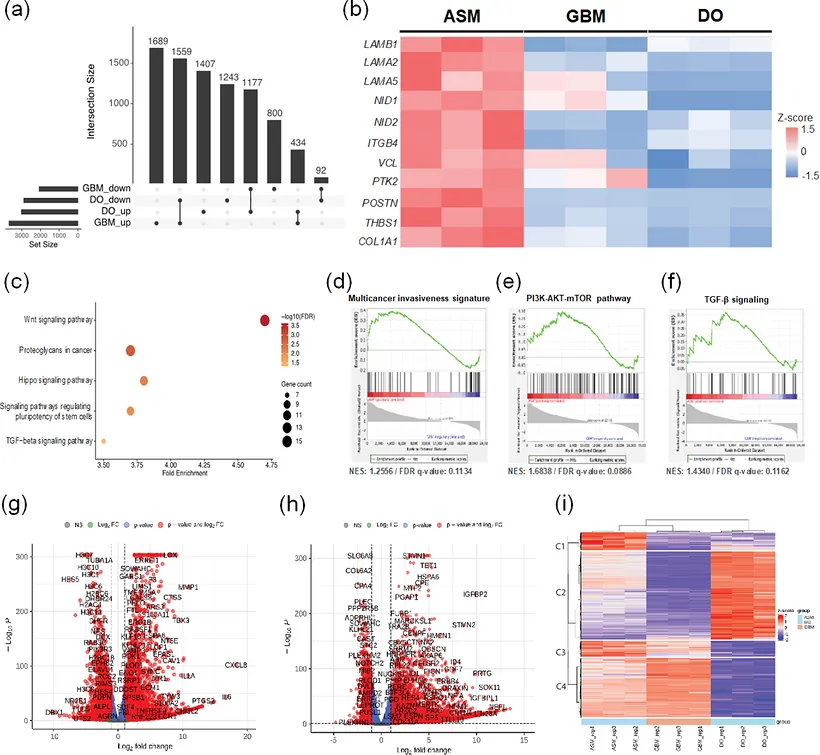
Transcriptional signaling pathway associated with the tumor‐brain organoid ASM formation. (a) UpSet plot showing the overlap of differentially expressed genes identified from pairwise comparisons between ASM, GBM spheroids, and DO organoids. (b) Heatmap of representative extracellular matrix (ECM), basement membrane, and focal adhesion‐related genes across ASM, GBM spheroids, and DO organoids. Color indicates normalized expression level across samples. (c) Pathway enrichment analysis of genes upregulated in ASM relative to both parental systems. Dot size indicates gene count, and color represents enrichment significance (−log_10_FDR). (d) GSEA comparing ASM and GBM spheroids showing enrichment of the multicancer invasiveness signature (GSEA Human Gene Set: M2572), (e) PI3K‐AKT‐mTOR signaling, and (f) TGF‐β signaling. Volcano plots of (g) DO versus ASM and (h) GBM versus ASM showing differentially expressed genes between experimental conditions. Each point represents a gene, plotted by log_2_ fold change versus −log_10_ adjusted *p*‐value. Genes meeting the differential expression criteria (adjusted *p*‐value < 0.05 and |log_2_FC| ≥ 1) are highlighted. (i) Heatmap showing the expression patterns of differentially expressed genes across ASM, DO, and GBM samples. Gene expression values are displayed as row‐scaled log_2_‐transformed TPM values.

To further resolve transcriptional differences among conditions, we compared Differential Gene Expression (DEG) profiles of ASMs with those of the two parental systems, GBM spheroids and DOs (Figure 5a). Intersection analysis of DEGs identified 1689 genes uniquely upregulated in ASMs relative to GBM spheroids and 1559 genes downregulated relative to DOs. Notably, 1177 genes were consistently upregulated in ASMs compared with both parental systems, indicating a distinct ASM‐specific transcriptional program, whereas 92 genes were commonly downregulated relative to both GBM and DO. These findings highlight molecular programs uniquely associated with ASM formation.

Genes upregulated in ASMs were strongly enriched for ECM‐ and adhesion‐related functions, consistent with the enhanced compaction and tumor‐organoid affinity observed phenotypically [79]. In particular, genes involved in laminin/basement membrane organization, focal adhesion, and ECM remodeling, including *LAMB1*, *LAMA2, LAMA5, NID1, NID2, ITGB4, VCL, PTK2, POSTN, THBS1*, and *COL1A1* [80, 81, 82], were more highly expressed in ASMs than in either GBM or DO alone (Figure 5b). These results support the interpretation that ASM formation is accompanied by induction of an ECM‐adhesion program that may promote integrin clustering, focal adhesion signaling, and physical stabilization at the tumor–host interface [80, 83].

Functional enrichment analysis further showed that genes consistently upregulated in ASMs were significantly enriched in pathways related to tumor–microenvironment interactions (Figure 5c), including the well‐established Wnt [84, 85], Hippo [86, 87], and TGF‐β signaling, as well as proteoglycan‐mediated ECM pathways [88]. Gene Set Enrichment Analysis (GSEA) comparing ASMs with GBM spheroids additionally demonstrated increased enrichment of the multicancer invasiveness signature and canonical oncogenic pathways, including PI3K‐AKT‐mTOR and TGF‐β signaling (Figure 5d–f). These pathway enrichments align with the observed phenotypic features of accelerated aggregation, adhesion, and invasion, suggesting that tumor‐host interactions drive coordinated transcriptional programs that support invasive behavior and structural integration within the ASM. Volcano plots (Figure 5g,h), the DEG heatmap (Figure 5i), and representative gene‐expression boxplots (Figure S4) further support this interpretation. Overall, these findings indicate that co‐culture with DOs promotes the transition of GBM toward an invasion‐competent state and that the ASM platform captures early tumor–host interactions not fully represented in GBM‐only spheroid models.

Subsequently, we generated GBM/5OA constructs and co‐cultured them with DOs using the same in vitro method, allowing for pre‐aggregation prior to implantation in the mouse subcutaneous space (Figure S5). GFP‐labeled GBM/5OA constructs were produced and were clearly distinguished from DOs (Figure S5a). Following this, the GBM/5OA constructs were implanted subcutaneously after 1 day of culture. Post‐mortem analysis after one month revealed a well‐organized structure in vivo (FigureS5b). In FigureS5c, H&E histological analysis showed aggressive GBM formation and integration with surrounding host tissue in the GBM/5OA group. However, the morphological structure of the DO was not observed. This outcome is likely due to the inability of DO to survive in the presence of GBM over the extended implantation period. Consequently, we explored alternative strategies to establish a stable GBM/5OA‐DO interface.

**FIGURE 6:**
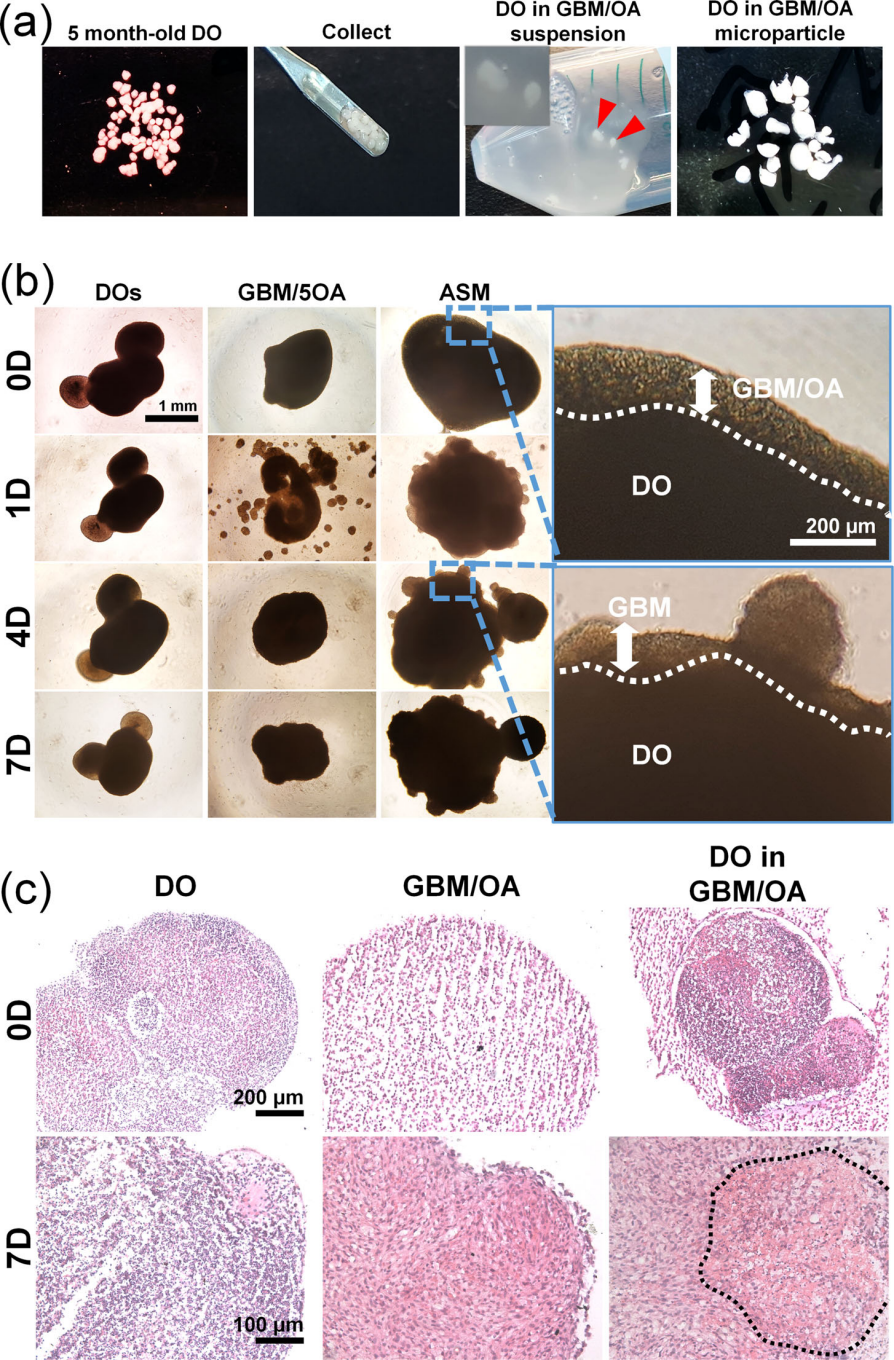
In vivo transplantation preparation of GBM/5OA and DO co‐culture. (a) Five‐month‐old DOs were collected and co‐cultured with the GBM/5OA suspension (red arrows). The DOs were then cross‐linked by dropwise addition into CaCl_2_ along with GBM/5OA. (b) Original DOs, GBM/5OA, and ASMs were cultured for 7 days. On day 0, GBM/5OA adhered well to the DOs. During culture, GBM cells were observed proliferating on the DO surface, released progressively from the degrading OA. (c) H&E analysis confirmed successful GBM integration into DOs by day 7, with evident infiltration beyond surface adhesion.

While our initial GBM/5OA microgel placement method enabled controlled interrogation of early interfacial interactions, we further developed a more scalable and uniform co‐culture approach to improve applicability for downstream applications, including in vivo implantation experiments (Figure S5). Specifically, we developed and implemented a direct and fast co‐culture method (Figure 6a). Most importantly, multiple DOs were immersed in a high‐density GBM/5OA suspension and subsequently crosslinked in CaCl_2_, thereby generating a defined, 3D “tumor nodule” shell surrounding the DOs. This structure recapitulates more closely an early‐stage tumor mass within a confined microenvironment than conventional approaches based on the addition of dissociated single tumor cells [89]. This core‐shell strategy provides several experimental advantages. First, it establishes a reproducible and pathologically relevant tumor‐host interface by ensuring a consistent initial tumor cell density and spatial relationship to the DO, which serves as the larger brain‐like tissue compartment. Second, the rapid crosslinking and tunable degradation of the 5OA matrix allow formation of a stable initial structure while subsequently permitting matrix loss, thereby mimicking a dynamic process analogous to extracellular matrix remodeling during early invasion. GBM cells became evenly distributed around the DO, and tissue integration became more pronounced over time (Figure 6b). The observed condensation suggests active cell‐mediated contraction and matrix remodeling, likely driven by GBM‐derived paracrine signaling that enhances tumor progression and invasion mimicry [17, 90]. Spontaneous GBM cell aggregation began as early as day 1. At this point in time, the ASM core‐shell displayed an irregular, zigzagging border. By day 4, this jagged morphology became more pronounced, and by day 7, the GBM shell had formed multiple small clusters on the DO surface. This pattern reflects clinical GBM behavior, where tumor cells collectively invade surrounding brain tissue, suggesting that the ASM model captures key biophysical and spatial features of early tumor dissemination [26]. This method ensures a high degree of reproducibility by establishing a consistent initial tumor cell density and pathologically relevant spatial arrangement to the DO, thereby reducing experimental variability. To further investigate tumorhost interactions, day 0 and day 7 samples were paraffin‐embedded, sectioned, and stained with H&E (Figure 6c). Histological analysis revealed that the initially well‐organized DO structure became increasingly disrupted over time. In contrast, GBM cells in 5OA, which appeared relatively dispersed on day 0, formed densely interconnected regions by day 7. In the core‐shell ASM, dense GBM tissue completely enveloped the DO, resulting in notable adverse effects. Apoptotic features were detected within the DO, including multiple nuclear fragments, eosinophilic necrotic debris, and vacuolated, enlarged cells, further indicating tumor‐induced cytotoxicity and progressive destruction of the host tissue architecture by the invading GBM [91].

This core–shell format of ASM model showed multiple advantages. First, by immersing multiple DOs into a high‐density GBM/5OA suspension followed by crosslinking in CaCl_2_, we created a defined, 3D tumor shell around the DO. This structure recapitulates an early‐stage tumor mass within its own micro‐environment, as opposed to the dispersed infiltration of single cells. Second, this fabrication strategy leverages the rapid crosslinking and tunable degradability of 5OA to establish an integrated tumor‐host interface. The subsequent, controlled degradation of the 5OA matrix actively mimics the tumor‐driven ECM remodeling that is a critical prerequisite for invasion. Finally, this method ensures a high degree of reliable production by establishing consistent initial tumor cell density and spatial positioning relative to the DO, thereby reducing experimental variability.

**FIGURE 7:**
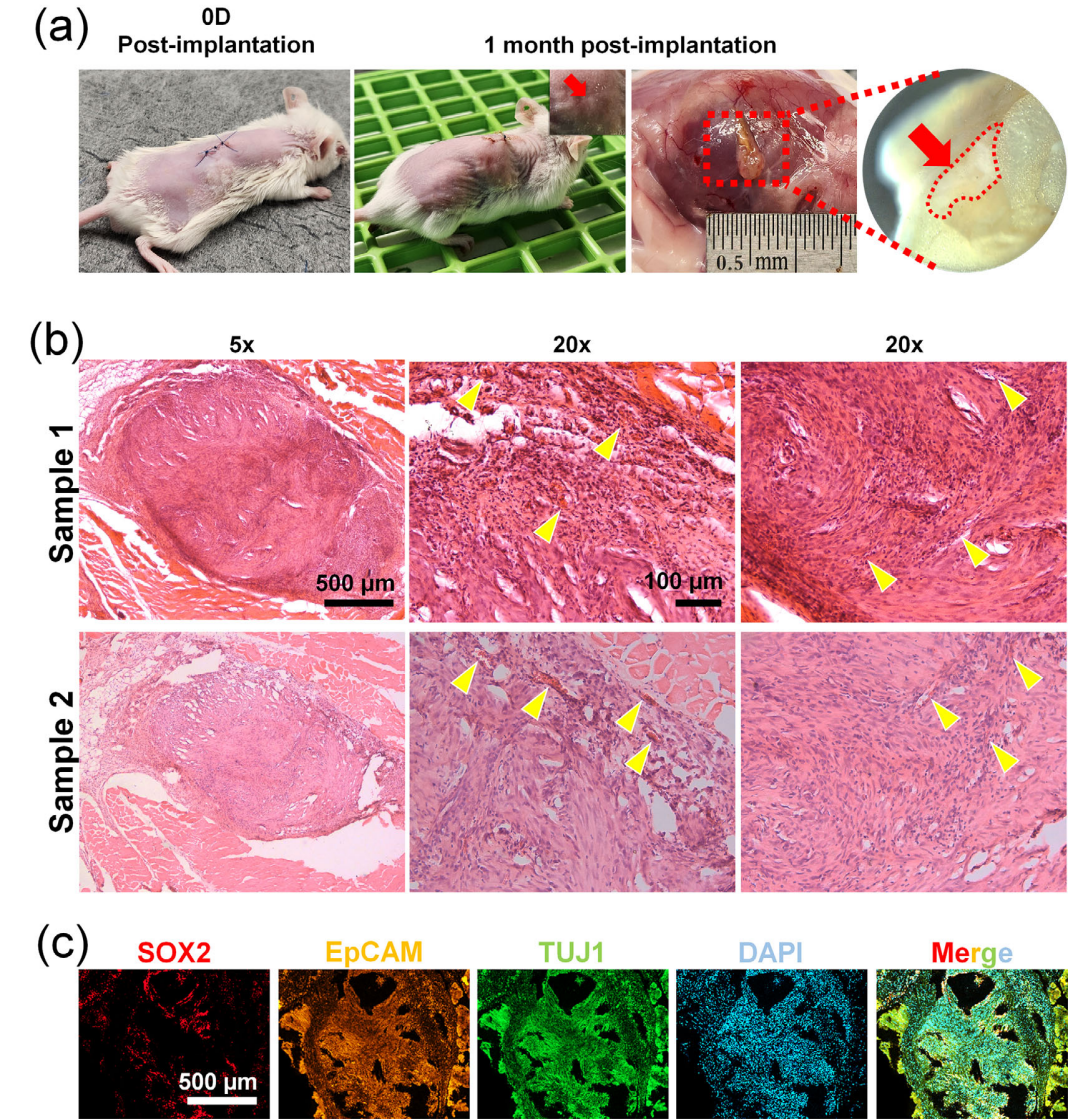
Subcutaneous implantation of GBM/5OA‐decorated DO constructs in the mouse subcutaneously. (a) A large volume of constructs was implanted immediately after instant fabrication. One month post‐implantation, the ASM was well organized and clearly visualized through gross view and stereomicroscope (b). H&E histological staining revealed a substantial GBM mass with evident vasculature infiltration (yellow arrows) within the newly formed tumor tissue. The original DO structure was no longer distinguishable. (c) Immunofluorescence analysis of the implanted tissue showed SOX2 (red), EpCAM (orange), TUJ1 (green), and DAPI (blue) nuclear counterstaining. SOX2^+^ tumor cells were distributed throughout the mass, indicating persistence of stem‐like proliferative compartments. EpCAM+ epithelial‐like clusters were interspersed within the tumor parenchyma, while TUJ1^+^ neuronal projections suggested host–tumor neural integration. The merged image demonstrates the spatial association of these markers, supporting the interpretation that implanted ASMs retain stem‐like features (SOX2), exhibit epithelial‐like remodeling (EpCAM), and display early signs of neurotropic interaction with host neural elements (TUJ1) in vivo.

To further assess the in vivo behavior of the DO‐GBM interface, we subcutaneously implanted the constructs into immunocompromised mice (Figure 7a). One month post‐implantation, analysis of the implantation site revealed the formation of newly developed tissue masses, as confirmed by gross morphology and stereomicroscopic imaging. H&E staining indicated the absence of distinguishable DO structures, instead revealing a large, densely packed mass of GBM cells (Figure 7b). Within this tumor mass, prominent infiltration of host vasculature (yellow arrows) was observed, suggesting successful biodegradation of the 5OA microgel, thereby enabling direct interaction between GBM cells and the host tissue microenvironment.

Immunofluorescence staining (Figure 7c) provided additional insights into the cellular composition and organization of the implanted tissue. SOX2 (red) staining identified widespread populations of stem‐like GBM cells, indicating sustained self‐renewal capacity. EpCAM (orange) staining revealed epithelial‐like clusters within the tumor parenchyma, reflecting phenotypic heterogeneity and evidence of epithelial remodeling. TUJ1 (green) staining identified neuronal processes extending toward and surrounding the tumor mass, consistent with early signs of host neurite outgrowth and potential attraction. These findings are consistent with the upregulation of invasion‐related genes in our RNA‐seq data, further confirming that this ASM model recapitulates key features of tumor invasiveness (Figure 4). DAPI (blue) nuclear counterstaining confirmed the high cellular density and the overall architecture of the tumor tissue. The spatial overlap of SOX2^+^, EpCAM^+^, and TUJ1^+^ regions at the invasion front suggests a heterogeneous tumor‐associated niche, characterized by aberrant co‐activation of stemness, neuronal, and epithelial transition, a potential hallmark of tumor‐induced plasticity hubs [48]. Collectively, these in vivo findings demonstrate that our 5OA‐based DO‐GBM ASM platform facilitates the rapid generation of tumor models that recapitulate key hallmarks of glioblastoma invasiveness and neurotropic behavior upon implantation.

In this study, we established a rapid and self‐organizing biofabrication platform using 5OA microgels to generate ASMs. This platform enables real‐time observation of early pathological interactions between mature brain organoids (e.g., DOs) and tumor components (e.g., GBM cells), providing a valuable tool for dissecting tumor‐–host dynamics during the earliest phases of malignancy. This platform successfully models key hallmarks of GBM progression, including tumor cell aggregation, extracellular matrix remodeling, formation of invasive fronts, and transcriptional reprogramming toward a mechanosensitive, invasion‐prone phenotype. The accelerated biodegradation of 5OA microgels enabled dynamic restructuring of GBM architecture and facilitated deep invasion into DOs, accompanied by the emergence of SOX2^+^/Vimentin^+^ invasive populations and nuclear condensation, which are indicative of high‐grade tumor aggressiveness. Transcriptomic profiling revealed upregulation of adhesion molecules (e.g., ITGA1, ITGA5), cytoskeletal regulators (e.g., CDC42, MYL9), and matrix components (e.g., LAMB1, LAMA2), alongside suppression of neuronal differentiation and synaptic genes. Enrichment of invasion‐associated pathways, including PI3K‐AKT‐mTOR and TGF‐β signaling, further supports the transition to an invasion‐competent state within the ASM microenvironment.

While the increased surface area of the DO compared to a control microgel could theoretically contribute to adhesion, the spatially restricted invasion, rapid transcriptional reprogramming, and evidence of cell‐cell interaction‐induced remodeling collectively indicate that active biochemical cues from the neural microenvironment, rather than passive physical adhesion, are the primary drivers of GBM malignancy in this system. Future studies using inert, size‐matched scaffolds will be required to formally disentangle the contributions of these variables.

In vivo transplantation studies corroborated these findings, demonstrating extensive GBM infiltration and host tissue remodeling, in contrast to the long‐term survival of DO‐only controls. These results underscore the destructive capacity of GBM cells even in ectopic environments and highlight the model's clinical relevance. Overall, the 5OA‐based ASM platform offers a physiologically relevant, scalable, and time‐efficient model for capturing the structural, molecular, and functional dynamics of early GBM invasion. It represents a promising preclinical tool for dissecting tumor‐microenvironment interactions in designated locations such as the brain and for accelerating therapeutic discovery targeting the earliest stages of glioblastoma dissemination. Yet, we reported that the inability of ASMs to sustain long‐term survival in vivo highlights the inherently aggressive and destructive nature of glioblastoma, even in ectopic implantation contexts. This observation reinforces the clinical relevance of our model system and its fidelity in capturing the malignant trajectory of GBM [92].

However, functional validation of certain pathways by protein‐level analysis (e.g., Western blot) represents an important direction for future studies. Moreover, further studies are required to elucidate the bidirectional biological interactions between GBM and neural organoid tissues in vivo. Future research should explore the mechanisms of cellular cooperation, immune evasion, and niche competition at the tumor–host interface. This study primarily employed the U87 model to validate the effectiveness of the 5OA ASM (co‐cultured DO‐GBM) platform. While this cell line serves as a reproducible canonical model for studying key aspects of GBM biology, GBM exhibits significant intertumoral heterogeneity. Future studies will be conducted to test this platform across diverse GBM models, including different cell lines, genetically distinct subtypes (e.g., IDH‐mutant, mesenchymal subtypes), and patient‐derived primary models for a comprehensive assessment of its generalizability and an exploration of how genetic background influences ASM behavior [10]. Such studies will enable a more comprehensive assessment of the platform's generalizability and provide insight into how tumor genetic background influences ASM behavior. In addition, the upregulation of integrin subunits (ITGA1, ITGA6), laminins (LAMB1), and the enrichment of PI3K‐AKT‐mTOR and TGF‐β signaling pathways in ASMs suggest these molecules as potential drivers of invasion. Future studies employing pathway‐specific inhibitors or genetic perturbations in the ASMs system will be essential to establish causal relationships and to identify the most critical nodes for therapeutic intervention. Such functional experiments are currently underway in our laboratory and will be reported in due course.

Moreover, while the subcutaneous xenograft model confirmed the aggressive nature of GBM and its ability to interact with host vasculature, the loss of identifiable DO structure underscores the limitations of an ectopic implantation site. To more faithfully model the brain microenvironment, future studies will employ orthotopic implantation into the mouse brain. In parallel, strategies to pre‑vascularize ASMs using engineered microvasculature could better support organoid survival and enable the study of angio‑invasive behaviors and blood–brain barrier interactions. Despite these challenges, our platform lays a solid foundation for advancing GBM research and holds potential to inform the development of next‐generation therapeutic strategies.

## 3 Conclusion

In summary, we have developed a bioengineered ASM platform that leverages the rapid self‐degradation of 5OA microgels to model the initial stages of glioblastoma invasion with high physiological relevance and temporal resolution. This system successfully recapitulates a sequence of critical events in early GBM pathogenesis, from rapid tumor cell migration, aggregation, EMT, and ECM remodeling to the establishment of invasive fronts populated by SOX2^+^/Vimentin^+^ cells. Moreover, transcriptomic and functional analyses confirmed that this process is driven by the upregulation of adhesion and mechanosensing pathways, which were facilitated via OA gel encapsulation during the first 2 days of culture, coupled with a suppression of neuronal programs. These observed host–tumor interactions collectively accelerate a shift of GBM toward a more aggressive, invasion‐competent state. The invasion and destructive phenotype of the resulting tumors was further validated by in vivo transplantation, which mirrored the aggressive trajectory of human GBM. Overall, our 5OA‐based ASM platform provides a scalable and time‐efficient tool that captures the structural, molecular, and functional complexity of early GBM invasion. It not only offers a novel preclinical avenue for dissecting tumor–microenvironment interactions but also establishes a robust foundation for future therapeutic screening aimed at preventing or interrupting the initial invasion of this lethal disease.

## 4 Experimental Section

### 4.1 Materials

Sodium alginate (Cat. No. A2033), MES hydrate (Cat. No. M8250, ≥99.5% (titration)), calcium chloride (CaCl_2_, 33.294 g, Cat. No. C5670), NaIO_4_ (Cat. No. 311 448, ACS reagent, ≥99.8%), NaCl (17.53 g, Cat. No. S9888, ACS reagent, ≥99.0%), ethylene glycol (Cat. No. 102 466), and D_2_O (Cat. No. 450 510) were purchased from Sigma‐Aldrich (St. Louis, MO, USA). Dry cellulose membrane dialysis bag (MD44, 3500 MW) was obtained from Labshark Co., Ltd. (Hunan, China). Steritop filters (Cat. No. SCGPS05RE) were purchased from Millipore GP Express (Billerica, MA, USA).

### 4.2 Preparation of 5OA Suspension and Generation of 5OA Microgels

OA was synthesized through an oxidation process as described in previous work, with some modifications [12]. Briefly, medium viscosity sodium alginate (10 g) was dissolved in 900 mL of ultrapure deionized water (diH_2_O) overnight for each condition. NaIO_4_ (0.540 g) was dissolved separately in 100 mL of diH_2_O and then added to the alginate solution to achieve a theoretical oxidation degree of 5%. This mixture was stirred vigorously in the dark (covered with aluminum foil) at room temperature for 24 h. Subsequently, MES hydrate (19.52 g) and NaCl (17.53 g) were added directly to the oxidized alginate solution (1 L), and the pH was adjusted to 6.5. Ethylene glycol (1 mL) was then added to halt the oxidation process. The OA macromer solution was purified using a dialysis membrane in diH_2_O for 5 days, with fresh diH_2_O being replaced twice daily. After dialysis, the OA macromer solution was filtered through bacteria‐excluding Steritop filters with a pore size of 0.22 µm under vacuum in a cell culture hood. Finally, the products were lyophilized for at least 10 days and stored at 4°C until use. For microgel generation, 5OA was reconstituted to 2% or 4% (w/v) by dissolving 100 or 200 mg of powder, respectively, in 5 mL sterile PBS (Cat. No. 70011–044, Thermo Fisher Scientific). The suspension of 5OA macromer in PBS was dropped directly into a bath containing the premade 150 mm CaCl_2_ crosslinker (details described below) to form spheroidal microgels. To maintain sterility, this process was performed under a laminar flow hood.

For crosslinking, a sterilized CaCl_2_ solution was used as a crosslinker for the OA/UA microgels. Briefly, 25 mm MES hydrate (9.762 g) was completely dissolved in 2 L of deionized water (diH_2_O). Subsequently, 150 mm CaCl_2_ (33.294 g) was added to the mixture, and the pH was adjusted to 7.2. The mixture was then filtered using bacteria‐excluding Steritop filters with a 0.22 µm pore size. Finally, 0.05 wt.% Tween 20 was added to the mixture. The prepared crosslinker was stored at room temperature until use.

### 4.3 Characterization of OA

The oxidized levels of UA and 1%, 2%, 4%, and 5% OA were quantified using IR spectroscopy combined with 2T2D‐COS. IR spectra were obtained using an iS50 FTIR spectrometer (ThermoFisher Scientific, USA) equipped with a built‐in ATR module (diamond crystal, 45° incident angle) and a DTGS detector at Kangwon Radiation Convergence Research Support Center of the Korea Basic Science Institute (KBSI), Kangwon National University. For each measurement, 256 scans were co‐added at a spectral resolution of 4 cm^−1^. Before performing the 2T2D‐COS analysis, all IR spectra underwent H_2_O spectrum subtraction, baseline correction, and area normalization using OriginPro 2026 (OriginLab Corporation, USA). The 2T2D correlation spectra were then generated using MATLAB R2025a (MathWorks, USA). The theoretical background and specific equations for 2T2D‐COS utilized in this study are based on the methodology reported by Noda et al. [93] and Jin et al. [32].

To confirm the successful synthesis of OA, 20 mg of UA, 1OA, 2OA, and 5OA were dissolved in 1 mL of D_2_O, a ^1^H‐nuclear magnetic resonance (^1^H‐NMR) spectrometer (Bruker Avance 400, Bruker Corporation, Billerica, MA, USA) was used to measure the chemical structure of OA at 25°C (*n* = 3). For the degradation test of 5OA, 2% and 4% 5OA were fabricated using the same crosslinking method. Microgel suspension (200 µL) was used to prepare around 20 microgels for each sample. Each sample was placed in a 15 mL conical tube in 15 mL Calcium ion/magnesium ion‐free PBS. PBS was changed daily after centrifugation, leaving 1 mL remaining in the tubes. Samples were washed three times with ddH_2_O, lyophilized and, the weight was measured at 0, 1, 3, and 5 days (*n* = 4).

### 4.4 Expansion of Human Glioblastoma Cells

U‐87 MG human glioblastoma cells (HTB‐14; ATCC, Manassas, VA, USA) were cultured in Dulbecco's Modified Eagle's Medium/Nutrient Mixture F12 (DMEM/F12; Cat. No.11320‐082, Thermo Fisher Scientific, Waltham, MA, USA) supplemented with 10% (v/v) fetal bovine serum (FBS; Cat. No. 26140079, Thermo Fisher Scientific) and 1% (v/v) penicillin‐streptomycin (P/S; Cat. No. GIB‐15140‐122, Thermo Fisher Scientific). Cells were maintained at 37°C in a humidified incubator with 5% CO_2_ and passaged every 3–4 days upon reaching approximately 80% confluence. For passaging, cells were washed once with PBS and incubated with 0.05% trypsin‐EDTA (Cat. No. 25200072, Thermo Fisher Scientific) for 3–5 min at 37°C until detachment. The cell suspension was neutralized with culture medium and collected by centrifugation at 300 × g for 5 min. The cell pellet was resuspended in the fresh culture medium for seeding. Cell counts were performed using a haemocytometer (HCCB, Livingstone) before encapsulation or further experiments.

### 4.5 Generation of DOs

DOs were generated using an established protocol [40] in combination with our previously describedmethod [94]. Human pluripotent stem cells between passage number 40 and 60, were cultured on hESC‐qualified Matrigel‐coated 6‐well plates (Cat. No. 325477, Corning Inc., Tewksbury, MA, USA) in supplemented Essential 8 Medium supplemented (Cat. No. A1517001, Thermo Fisher Scientific). When the cells reached approximately 80% confluency, they were dissociated into single cells using Accutase (Cat. No. 07922, STEMCELL Technologies). Approximately 1000 cells were seeded into each microwell of AggreWell^TM^800 6‐well plates (Cat. No. 34421, STEMCELL Technologies) in AggreWellTM EB Formation Medium (Cat. No. 05893, STEMCELL Technologies) supplemented with 10 µm ROCK inhibitor Y‐27632 (Cat. No. Y0503, Sigma‐Aldrich) to promote embryoid body (EB) formation. After 24 h, EBs were collected and transferred to ultra‐low attachment 60 mm dishes (Cat. No.CLS4615, Sigma‐Aldrich) in organoid medium composed of Neurobasal‐A medium (Cat. No. 108880222, Thermo Fisher Scientific), B‐27 supplement minus vitamin A (Cat. NO. 12587001, Thermo Fisher Scientific), GlutaMAX (1:100, Cat. No. 35050) and penicillin‐streptomycin (1:100). For neural induction, organoid medium was supplemented with 2.5 µm dorsomorphin (Cat. No. P5499, Sigma‐Aldrich), 10 µm SB‐431542 (Cat.No. 1614, Tocris). Media were refreshed daily duringthe first 5 days. From day 6 to day 24, organoids were maintained in organoid medium supplemented with c20 ng/mL epidermal growth factor (EGF; Cat. No. 236‐EG; R&D Systems) and 20 ng/mL basic fibroblast growth factor (bFGF; Cat. No. 233‐FB, R&D Systems). From day 24 and day 43, organoids were maintained in organoid medium supplemented with t20 ng/ml brain‐derived neurotrophic factor (BDNF; Cat. No. 450‐02, Peprotech) and 20 ng/ml neurotrophin‐3 (NT3;Cat. No. 450‐03, Peprotech). Thereafter, organoids were maintained in organoid medium without additional growth factors, with medium changed every four days. Organoids were matured for at least 43 days in culture before use in experiments, including co‐culture formation.

### 4.6 Generation of the ASM Model

U87 GBM cells were harvested, centrifuged at 1800 rpm for 5 min, and resuspended in either 2% or 4% OA macromer at a density of 1.5 × 10^8^ cells/mL. To preserve cell integrity, the cell suspension was gently mixed without repeated pipetting. The cell–5OA suspension was loaded into a syringe fitted with an 18G needle and dispensed dropwise through a 30G needle into a sterile‐filtered 150 mm CaCl_2_ solution to initiate crosslinking and generate microgels. Approximately 500 µL of suspension yielded 40–50 microgels. Microgels were transferred individually into wells of U‐bottom 96‐well plates (Cat. No. 7007, Corning Inc.) containing 200 µL of DMEM supplemented with 10% FBS and 5% P/S. The medium was refreshed daily by gentle partial replacement (150 µL). To create ASMs, individual GBM/5OA microgels were placed in direct contact with mature DOs in ultra‐low attachment 96‐well plates (Cat. No. 7007, Corning Inc.). Constructs were co‐cultured in DMEM supplemented with 10% FBS and 5% P/S, and brightfield images were taken at 0, 24, 48, and 72 h post‐assembly.

### 4.7 Histological Analysis and Immunofluorescence Staining

Cultured samples were sectioned using the paraffin embedding method described in previous work [12, 95]. Paraffin‐embedded GBM aggregate and ASM sections were processed for immunofluorescence staining. Briefly, sections were deparaffinized in xylene and rehydrated through a graded ethanol series. Antigen retrieval was performed in citrate buffer (10 mm sodium citrate, pH 6.0) using heat‐induced epitope recovery. After cooling to room temperature, slides were rinsed in PBS and permeabilized with 0.5% Triton X‐100 (Cat. No. X100, Sigma‐Aldrich) in PBS for 10 min. Non‐specific binding was blocked with 5% (w/v) bovine serum albumin (BSA; Cat. No. A9647, Sigma‐Aldrich) in PBS for 1 h at room temperature on a shaker.

Primary antibodies were diluted in 1% BSA/PBS and incubated overnight at 4°C, using SOX2 (rabbit polyclonal, 1:500, Cat. No. AB5603, Sigma‐Aldrich) and Vimentin (rabbit monoclonal, 1:1500, Cat. No. 9856, Cell Signaling Technology). SOX2 and Vimentin were detected with Alexa Fluor 488‐conjugated and Alexa Fluor 594‐conjugated secondary antibody (1:200, Thermo Fisher Scientific). Nuclei were counterstained with 4′,6‐diamidino‐2‐phenylindole (DAPI; 1:1000, Cat. No. D1306, Thermo Fisher Scientific) for 15 min. Slides were washed in PBS, mounted with antifade medium, and imaged on an Olympus FV3000 laser scanning confocal microscope at 20× magnification. For live‐cell imaging, samples were monitored using an IncuCyte SX5 (Sartorius, Bohemia, NY, USA) at 4× magnification. Brightfield images were acquired every 2 h over the course of the assay. Image sequences were exported and analyzed in Fiji (ImageJ). The initial spheroid centroid at Day 0 was defined as the reference point. Cellular dispersal was quantified as the mean radial displacement of cells from this centroid over time. Distances were calculated in pixels and converted to physical units using system calibration (2.83 µm per pixel). For each time point, displacement values were averaged across biological replicates (*n* = 9). Linear regression analysis was performed in R (v4.2.2) to estimate displacement rates over time. A significantly greater rate of displacement was observed in GBM‐DO ASMs compared to GBM cultured alone (interaction F(1,68) = 104.7, *p* < 0.001). Data were created with BioRender and quantitative data were visulaized in R.

### 4.8 Live/Dead Cytotoxicity Assay

To evaluate the cytotoxicity of 5OA independent of tumor effects, DOs were cultured under identical conditions either with or without 5OA encapsulation. Mature DOs were transferred to ultra‐low attachment plates (Cat. No. 7007, Corning Inc.) and assigned to either a 5OA‐encapsulated group or an untreated control group. Both groups were maintained for 5 days in the same medium used for ASM formation.

Viability was assessed using a Live/Dead Viability/Cytotoxicity Kit (Cat. No. L3224, Thermo Fisher Scientific) according to the manufacturer's protocol. Calcein‐AM was used to label viable cells in green, and ethidium homodimer‐1 was used to label dead cells in red. Fluorescence images were acquired using an EVOS M3000 microscope (Thermo Fisher Scientific) at 4× magnification under identical exposure settings.

Image analysis was performed in FIJI (ImageJ). For each organoid, the entire organoid area was selected as the region of interest, and background fluorescence was measured from adjacent cell‐free regions. Corrected integrated density (Corrected IntDen) was calculated as integrated density − (organoid area × mean background fluorescence). Three biological replicates were analyzed per condition (*n* = 3). Statistical analyses were performed in R (v4.2.2). Normality and homogeneity of variance were assessed using the Shapiro–Wilk and Levene's tests, respectively, and group comparisons were performed using an unpaired two‐tailed Student's *t*‐test. Data are presented as mean ± SD, and *p *< 0.05 was considered statistically significant.

### 4.9 RNA Sequencing and Analysis

Total RNA was extracted from samples collected on day 4 using the RNeasy Micro Kit (Cat. No. 74004, Qiagen, Toronto, Canada) according to the manufacturer's protocol. RNA quantity and purity were assessed using a NanoDrop 8000 spectrophotometer (Cat. No. ND‐8000‐GL, Thermo Fisher Scientific). RNA samples were sent to Macrogen (Seoul, Republic of Korea) for library preparation and sequencing. Downstream data processing, including quality control, alignment, and differential expression analysis, was performed in RStudio (R.4.4.2) using standard Bioconductor packages. For the control brain organoid versus GBM/5OA comparison, public control data were obtained from GSE248479. Because only DESeq2‐normalized counts were available from GEO, we generated DESeq2‐normalized counts for our dataset and then applied quantile normalization across the combined matrix to harmonize distributions for cross‐cohort visualization and effect‐size comparisons. For the GBM/5OA versus ASM analysis, raw gene‐level counts were filtered to protein‐coding genes, with chrY and duplicated symbols removed; genes with ≥10 counts in ≥3 samples were retained. Differential expression was performed with DESeq2 (version 1.46.0) using median‐ratio normalization, dispersion shrinkage, and Wald tests; significance was set at FDR < 0.05 with |log2FC| ≥ 1. Results were visualized with EnhancedVolcano (version 1.24.0), and selected signatures were displayed with ComplexHeatmap (version 2.22.0). Pathway‐level changes were evaluated using Gene Set Enrichment Analysis (GSEA), performed with the GSEA desktop application (Broad Institute, Cambridge, MA) on macOS. The weighted enrichment statistic was applied, with genes ranked by the Signal2Noise metric in descending order. Gene sets from MSigDB were queried against selected Hallmark, KEGG, and Gene Ontology biological process (GO‐BP) collections, with a minimum gene set size of 15 and a maximum of 500. Probe sets were collapsed to a single gene using the Max_probe method, and normalization was performed using the meandiv mode. Features with no symbol match were omitted.

### 4.10 Animal Studies

All animal experiments complied with ARRIVE (Animal Research: Reporting of In Vivo Experiments) guidelines. Studies also carried out in accordance with the Guidance on the operation of the Animals (Scientific Procedures) Act 1986 and associated guidelines and the NIH (National Research Council) Guide for the Care and Use of Laboratory Animals. All animal studies were conducted under the animal research protocol (No. 22–268) approved by the Committee on the Use of Live Animals in Teaching and Research (CULATR) at the University of Hong Kong. The studies adhered to the Animals (Control of Experiments) Ordinance (Hong Kong) and guidelines from the Centre for Comparative Medical Research (CCMR), Li Ka Shing Faculty of Medicine, The University of Hong Kong.

The NOD.Cg‐Prkdc^scid^Il2rg^tm1Wjl^/SzJ mouse strain, commonly known as NSG mice (4–6 weeks old, male), was used for the experiments. The mice were obtained from the Experimental Animal Center of the University and had access to food and water ad libitum, and were maintained under pathogen‐free conditions. All procedures were performed under anesthesia using intraperitoneal injections of a ketamine/xylazine mixture (100 mg/kg ketamine [Cat. No. 013004, AlfaMedic Ltd.] + 10 mg/kg xylazine [Cat. No. 013006, AlfaMedic Ltd.]). Anesthesia was confirmed by checking body reflexes, and eye ointment (Duratears ointment, Cat. No. 05686, Alcon, Fort Worth, TX, USA) was applied to prevent blindness. The subcutaneous transplantation sites on the mice were decontaminated with alternating applications of povidone‐iodine (Betadine) and 70% alcohol using sterile cotton swabs. The animals were administered buprenorphine (∼0.1 mg/kg) 30–60 min before surgery and twice a day for 3 days thereafter.

### 4.11 In Vivo Procedure

To confirm the rapid remodeling of the DO‐GBM, in vivo transplantation was performed using DOs and GBM/5OA. The GBM cells were prepared as usual, followed by the loading of 5OA macromer using the same procedures outlined above. Before transplantation, the dorsal subcutaneous area was divided into left and right, and the transplantation was performed. One month post‐implantation, the mice were euthanized with an overdose of pentobarbital (250 mg/kg) (Dorminal, Cat. No. 013003, AlfaMedic Ltd.), administered intraperitoneally. Samples and tissues were harvested. Transplants were fixed in 10% normal buffered formalin for 24 h before embedding, while day 0 samples were kept in 70% ethanol as a control.

### 4.12 Histological Analysis

A stereoscope was used to acquire gross images of the harvested samples after fixation in 70% ethanol. All samples were dehydrated using a gradient of ethanol (from 70% to 100%) and underwent a series of treatments with ethanol, xylene‐ethanol, and xylene, each lasting 30 min. Subsequently, all samples were embedded in paraffin and sectioned to a thickness of 12 µm using a rotary microtome (Cat. No. Leica RM2155, Leica Microsystems, Wetzlar, Germany). The sections were deparaffinized, rehydrated, and stained with hematoxylin (Cat. No. SH4777, Harris Hematoxylin, Cancer Diagnostic Inc., Durham, NC, USA) and eosin (Cat. No. CS701, Dako, Glostrup, Denmark) (H&E).

### 4.13 Statistical Analysis

All quantitative data were presented as mean ± standard deviation (SD) unless otherwise stated in the figure legend. For each experiment, the sample size (*n*) equals three unless explicitly indicated. A *p*‐value of less than 0.05 was considered statistically significant (*p < 0.05, **p < 0.01, ***p < 0.001).

For the comparison of gene expression levels between groups (e.g., DO‐GBM ASMs vs. GBM/5OA spheroids, DO‐GBM ASMs vs. DO), statistical significance was assessed using a Benjamini–Hochberg adjustment. For RNA‐sequencing data analysis, genes with expression counts across all samples less than 10 were filtered out prior to differential expression testing. Differential expression analysis across samples was performed using DESeq2 (version 1.46.0) based on a two‐sided Wald test. *P*‐values were adjusted for multiple testing using the Benjamini–Hochberg procedure to control the false discovery rate (FDR). Genes with an adjusted *p‐*value (padj) < 0.05 and an absolute log_2_ fold change (|log_2_FC|) ≥ 1 were considered significantly differentially expressed. All statistical tests were two‐sided. No adjustments were made for multiple comparisons unless explicitly stated. Statistical analyses and graphical representations were generated using BioRender and R Studio (2024.04.2).

## Author Contributions

C.L., H.R.H., S.C. (Shihui Chen), and W.Y.C contributed equally to this work as co‐first authors. S.J.L. and A.N.C. contributed equally to this work as co‐corresponding authors. S.J.L. and A.N.C. conceptualized the research idea and designed the project. C.L., H.R.H., and W.Y.C performed the experiments and established the DO‐GBM ASM model, analyzed the data, and drafted the manuscript. W.Y.C. conducted RNA sequencing analysis. S.C. (Shihui Chen) contributed to brain organoid generation, conducted live‐dead assay, and manuscript writing. S.C. (Summer Cao) supported the initial stage of brain organoid generation and GBM protocol optimization. K.Z. contributed to immunofluorescence staining and confocal microscopy. A.C. guided and contributed to confocal imaging. M.K. provided pharmacological reagents and provided assay interpretation. F.D. provided genetic experimental resources and provided an intellectual molecular pathway. Y.P and Y.M.J provided IR data with its analysis. Y.D.K provided experimental resources. S.J.L. and A.N.C. are the senior authors who supervised the study and provided instruction, design suggestions for the improvement of the experiments. All authors provided comments and corrections to the manuscript writing.

## Conflicts of Interest

The authors declare no conflicts of interest.

## Supporting information




**Supporting File 1**: adhm71231‐sup‐0001‐SuppMat.docx.

**Supporting File 2**: adhm71231‐sup‐0002‐MovieS1.mp4.

**Supporting File 3**: adhm71231‐sup‐0003‐MoviesS2.mp4.

## Data Availability

The datasets supporting the conclusions of this article are available in the GEO database (GSE325111).

## References

[1] K. Vigneswaran , S. Neill , and C. G. Hadjipanayis , “Beyond the World Health Organization Grading of Infiltrating Gliomas: Advances in the Molecular Genetics of Glioma Classification,” Annals of Translational Medicine 3 (2015): 95. 26015937 10.3978/j.issn.2305-5839.2015.03.57 PMC4430738

[2] C. Horbinski , T. Berger , R. J. Packer , and P. Y. Wen , “Clinical Implications of the 2021 Edition of the WHO Classification of Central Nervous System Tumours,” Nature Reviews Neurology 18 (2022): 515–529, 10.1038/s41582-022-00679-w. 35729337

[3] K. M. Hotchkiss , P. Karschnia , K. C. Schreck , et al., “A Brave New Framework for Glioma Drug Development,” Lancet Oncology 25 (2024): e512–e519, 10.1016/S1470-2045(24)00190-6. 39362262 PMC11983286

[4] D. N. Louis , A. Perry , G. Reifenberger , et al., “The 2016 World Health Organization Classification of Tumors of the central Nervous System: A Summary,” Acta Neuropathologica 131 (2016): 803–820, 10.1007/s00401-016-1545-1. 27157931

[5] A. Omuro and L. M. DeAngelis , “Glioblastoma and Other Malignant Gliomas,” Jama 310 (2013): 1842–1850, 10.1001/jama.2013.280319. 24193082

[6] P. J. Kelly , “Gliomas: Survival, Origin and Early Detection,” Surgical Neurology International 1 (2010): 96, 10.4103/2152-7806.74243. 21246059 PMC3019361

[7] D. Crosby , S. Bhatia , K. M. Brindle , et al., “Early Detection of Cancer,” Science 375 (2022): aay9040, 10.1126/science.aay9040. 35298272

[8] A. M. Alsharabasy and A. Pandit , “Hyaluronan‐Based Hydrogels for 3d Modeling of Tumor Tissues,” Tissue Engineering Part C: Methods 30 (2024): 452–499, 10.1089/ten.tec.2024.0271. 39345138

[9] Q. Fan , H. Wang , T. Gu , et al., “Modeling the Precise Interaction of Glioblastoma With Human Brain Region‐Specific Organoids,” Iscience 27 (2024): 109111, 10.1016/j.isci.2024.109111. 38390494 PMC10882168

[10] M. Chadwick , C. Yang , L. Liu , et al., “Rapid Processing and Drug Evaluation in Glioblastoma Patient‐Derived Organoid Models With 4D Bioprinted Arrays,” Iscience 23 (2020): 101365. 32731171 10.1016/j.isci.2020.101365 PMC7393526

[11] V. G. Prior , S. Maksour , S. Miellet , et al., “Parsing the Effect of co‐Culture With Brain Organoids on Diffuse Intrinsic Pontine Glioma (DIPG) Using Quantitative Proteomics,” International Journal of Biochemistry & Cell Biology 174 (2024): 106617, 10.1016/j.biocel.2024.106617. 39009182

[12] S. J. Lee , O. Jeon , Y. B. Lee , et al., “In Situ Cell Condensation‐Based Cartilage Tissue Engineering via Immediately Implantable High‐Density Stem Cell Core and Rapidly Degradable Shell Microgels,” Advanced Composites and Hybrid Materials 8 (2025): 1–14, 10.1007/s42114-025-01406-x.

[13] J. Lee , O. Jeon , J. Koh , et al., “Micromechanical Property Mismatch Between Pericellular and Extracellular Matrices Regulates Stem Cell Articular and Hypertrophic Chondrogenesis,” Matter 6 (2023): 475–492.

[14] C. Liang , S. Wu , Z. Huang , et al., “Harnessing Oxidized Alginate Microgels for Rapid and Self‐Assembling Dental Tissue Organogenesis In Vitro and In Vivo,” Small Science 5 (2025): 202500053, 10.1002/smsc.202500053. PMC12697820 41395504

[15] W. Wang , R. Wen , Q. Li , Z. Cai , Z. Ou , and L. Zheng , “Leveraging the Mononuclear Phagocyte System for Advancing Glioma Theranostics: Current Insights and Future Directions,” Aggregate 6 (2025): 70182.

[16] P. Suarez‐Meade , R. Whitehead , S. Rosenfeld , P. Schiapparelli , K. Konstantopoulos , and A. Quinones‐Hinojosa , “Extracellular Matrix Stiffness Conditions Glioblastoma Cells for Long‐Term Migration: Mechanical Memory as a Driver of Invasion and Recurrence in Glioblastoma,” Neuro‐Oncology 28 (2025): 19–37. 10.1093/neuonc/noaf205 PMC12962637 40973065

[17] V. P. Ferrer , V. M. Neto , and R. Mentlein , “Glioma Infiltration and Extracellular Matrix: Key Players and Modulators,” Glia 66 (2018): 1542–1565, 10.1002/glia.23309. 29464861

[18] S. Kang , M. E. Ughetta , J. Y. Zhang , et al., “Glioblastoma Shift From Bulk to Infiltrative Growth Is Guided by Plexin‐B2‐Mediated Microglia Alignment in Invasive Niches,” Nature Cancer 6 (2025): 1505–1523, 10.1038/s43018-025-00985-4. 40442367 PMC12353550

[19] K. M.‐Y. Kiang , W. Tang , Q. Song , et al., “Targeting Unfolded Protein Response Using Albumin‐Encapsulated Nanoparticles Attenuates Temozolomide Resistance in Glioblastoma,” British Journal of Cancer 128 (2023): 1955–1963, 10.1038/s41416-023-02225-x. 36927978 PMC10147657

[20] K. M. Kiang , Y. Shen , Y. K. H. Wong , et al., “1, 25D3 Reprograms Mitochondrial Quality Control via Sirtuin and Sensitizes Glioblastoma to Chemotherapy,” Molecular Therapy (2026), 10.1016/j.ymthe.2026.03.006. PMC13330008 41792999

[21] V. Fedorova , V. Pospisilova , T. Vanova , et al., “Glioblastoma and Cerebral Organoids: Development and Analysis of an In Vitro Model for Glioblastoma Migration,” Molecular Oncology 17 (2023): 647–663, 10.1002/1878-0261.13389. 36744875 PMC10061278

[22] J. Kim , R. Kim , W. Lee , et al., “Assembly Of Glioblastoma Tumoroids And Cerebral Organoids: A 3D In Vitro Model For Tumor Cell Invasion,” Molecular Oncology 19 (2025): 698–715, 10.1002/1878-0261.13740. 39473365 PMC11887666

[23] E. Rosén , H. B. Mangukiya , L. Elfineh , et al., “Inference of Glioblastoma Migration and Proliferation Rates Using Single Time‐Point Images,” Communications Biology 6 (2023): 402. 37055469 10.1038/s42003-023-04750-0 PMC10102065

[24] S. Sarkar , J. Greer , N. J. Marlowe , et al., “Stemness, Invasion, and Immunosuppression Modulation in Recurrent Glioblastoma Using Nanotherapy,” WIREs Nanomedicine and Nanobiotechnology 16 (2024): 1976, 10.1002/wnan.1976. 39091260

[25] Q. Wang , B. Hu , X. Hu , et al., “Tumor Evolution of Glioma‐Intrinsic Gene Expression Subtypes Associates With Immunological Changes in the Microenvironment,” Cancer Cell 32 (2017): 42–56.e6, 10.1016/j.ccell.2017.06.003. 28697342 PMC5599156

[26] K. J. Wolf , J. Chen , J. D. Coombes , M. K. Aghi , and S. Kumar , “Dissecting and Rebuilding the Glioblastoma Microenvironment With Engineered Materials,” Nature Reviews Materials 4 (2019): 651–668, 10.1038/s41578-019-0135-y. PMC7347297 32647587

[27] A. C. Greenwald , N. G. Darnell , R. Hoefflin , et al., “Integrative Spatial Analysis Reveals a Multi‐Layered Organization of Glioblastoma,” Cell 187 (2024): 2485–2501.e26, 10.1016/j.cell.2024.03.029. 38653236 PMC11088502

[28] F. Jacob , G.‐L. Ming , and H. Song , “Generation and Biobanking of Patient‐Derived Glioblastoma Organoids and Their Application in CAR T Cell Testing,” Nature Protocols 15 (2020): 4000–4033, 10.1038/s41596-020-0402-9. 33169003

[29] W. D. Pontius , L. C. Wallace , K. Fife , and C. G. Hubert , “Human Glioblastoma Organoids to Model Brain Tumor Heterogeneity Ex Vivo,” Brain Tumors 158 (2020): 133–158.

[30] C. G. Gomez , M. Rinaudo , and M. A. Villar , “Oxidation of Sodium Alginate and Characterization of the Oxidized Derivatives,” Carbohydrate Polymers 67 (2007): 296–304, 10.1016/j.carbpol.2006.05.025.

[31] J.‐S. Yang , Y.‐J. Xie , and W. He , “Research Progress on Chemical Modification of Alginate: A Review,” Carbohydrate Polymers 84 (2011): 33–39, 10.1016/j.carbpol.2010.11.048.

[32] S. Jin , S. V. Reverdatto , V. V. Ermolenkov , A. Shekhtman , Y. M. Jung , and I. K. Lednev , “Encapsulation of mRNA in Therapeutics like Lipid Nanoparticles Probed by Deep‐UV Resonance Raman Spectroscopy,” Analytical Chemistry 98 (2026): 3523–3530, 10.1021/acs.analchem.5c04246. 41537655 PMC12903059

[33] G. Lawrie , I. Keen , B. Drew , et al., “Interactions Between Alginate and Chitosan Biopolymers Characterized Using FTIR and XPS,” Biomacromolecules 8 (2007): 2533–2541, 10.1021/bm070014y. 17591747

[34] W. Rajapaksha , I. H. Nicholas , T. Thoradeniya , D. N. Karunaratne , and V. Karunaratne , “Novel Alginate Nanoparticles for the Simultaneous Delivery of Iron and Folate: A Potential Nano‐Drug Delivery System for Anaemic Patients,” RSC Pharmaceutics 1 (2024): 259–271, 10.1039/D3PM00068K.

[35] K. A. Kristiansen , H. B. Tomren , and B. E. Christensen , “Periodate Oxidized Alginates: Depolymerization Kinetics,” Carbohydrate Polymers 86 (2011): 1595–1601, 10.1016/j.carbpol.2011.06.069.

[36] K. H. Bouhadir , K. Y. Lee , E. Alsberg , K. L. Damm , K. W. Anderson , and D. J. Mooney , “Degradation of Partially Oxidized Alginate and Its Potential Application for Tissue Engineering,” Biotechnology Progress 17 (2001): 945–950, 10.1021/bp010070p. 11587588

[37] S. Reakasame and A. R. Boccaccini , “Oxidized Alginate‐Based Hydrogels for Tissue Engineering Applications: A Review,” Biomacromolecules 19 (2018): 3–21, 10.1021/acs.biomac.7b01331. 29172448

[38] A.‐N. Cho , Y. Jin , Y. An , et al., “Microfluidic Device With Brain Extracellular Matrix Promotes Structural and Functional Maturation of Human Brain Organoids,” Nature Communications 12 (2021): 4730, 10.1038/s41467-021-24775-5. PMC8342542 34354063

[39] J. Brás , D. Henriques , R. Moreira , et al., “Establishment and Characterization of Human Pluripotent Stem Cells‐Derived Brain Organoids to Model Cerebellar Diseases,” Scientific Reports 12 (2022): 12513, 10.1038/s41598-022-16369-y. 35869235 PMC9307606

[40] F. Birey , J. Andersen , C. D. Makinson , et al., “Assembly of Functionally Integrated Human Forebrain Spheroids,” Nature 545 (2017): 54–59, 10.1038/nature22330. 28445465 PMC5805137

[41] S. Zhang , L. Yuan , P. Lin , et al., “Cancer Neuroscience: Signaling Pathways and New Therapeutic Strategies for Cancer,” Signal Transduction and Targeted Therapy 11 (2026): 66, 10.1038/s41392-025-02364-y. 41724757 PMC12926231

[42] D. Hendriks , A. Pagliaro , F. Andreatta , et al., “Human Fetal Brain Self‐Organizes Into Long‐Term Expanding Organoids,” Cell 187 (2024): 712–732.e38, 10.1016/j.cell.2023.12.012. 38194967

[43] K. Reiss and P. Saftig , “The “a Disintegrin and Metalloprotease”(ADAM) Family of Sheddases: Physiological and Cellular functions,” in Seminars in Cell & Developmental Biology (Elsevier, 2009), 126–137. 10.1016/j.semcdb.2008.11.002 19049889

[44] S. Zhong and R. A. Khalil , “A Disintegrin and Metalloproteinase (ADAM) and ADAM With Thrombospondin Motifs (ADAMTS) Family in Vascular Biology and Disease,” Biochemical Pharmacology 164 (2019): 188–204, 10.1016/j.bcp.2019.03.033. 30905657 PMC6580420

[45] E.‐J. Lim , J.‐H. Kang , Y.‐J. Kim , S. Kim , and S.‐J. Lee , “ICAM‐1 Promotes Cancer Progression by Regulating SRC Activity as an Adapter Protein in Colorectal Cancer,” Cell Death & Disease 13 (2022): 417, 10.1038/s41419-022-04862-1. 35487888 PMC9054780

[46] Z. Qiu , Y. Wang , Z. Zhang , et al., “Roles of Intercellular Cell Adhesion Molecule‐1 (ICAM‐1) in Colorectal Cancer: Expression, Functions, Prognosis, Tumorigenesis, Polymorphisms and Therapeutic Implications,” Frontiers in Oncology 12 (2022): 1052672, 10.3389/fonc.2022.1052672. 36505809 PMC9728583

[47] A. Benedicto , I. Romayor , and B. Arteta , “Role of Liver ICAM‐1 in Metastasis,” Oncology Letters 14 (2017): 3883–3892, 10.3892/ol.2017.6700. 28943897 PMC5604125

[48] M. I. De Silva , B. W. Stringer , and C. Bardy , “Neuronal and Tumourigenic Boundaries of Glioblastoma Plasticity,” Trends in Cancer 9 (2023): 223–236, 10.1016/j.trecan.2022.10.010. 36460606

[49] Y. A. Yabo , S. P. Niclou , and A. Golebiewska , “Cancer Cell Heterogeneity and Plasticity: A Paradigm Shift in Glioblastoma,” Neuro‐Oncology 24 (2022): 669–682, 10.1093/neuonc/noab269. 34932099 PMC9071273

[50] A. Linkous , D. Balamatsias , M. Snuderl , et al., “Modeling Patient‐Derived Glioblastoma With Cerebral Organoids,” Cell Reports 26 (2019): 3203–3211.e5, 10.1016/j.celrep.2019.02.063. 30893594 PMC6625753

[51] M. J. Rybin , M. E. Ivan , N. G. Ayad , and Z. Zeier , “Organoid Models of Glioblastoma and Their Role in Drug Discovery,” Frontiers in Cellular Neuroscience 15 (2021): 605255, 10.3389/fncel.2021.605255. 33613198 PMC7892608

[52] X. Wang , Y. Sun , D. Y. Zhang , G.‐L. Ming , and H. Song , “Glioblastoma Modeling With 3D Organoids: Progress and Challenges,” Oxford Open Neuroscience 2 (2023): kvad008. 38596241 10.1093/oons/kvad008 PMC10913843

[53] C. Wang , M. Sun , C. Shao , et al., “A Multidimensional Atlas of Human Glioblastoma‐Like Organoids Reveals Highly Coordinated Molecular Networks and Effective Drugs,” NPJ Precision Oncology 8 (2024): 19, 10.1038/s41698-024-00500-5. 38273014 PMC10811239

[54] N. Skarne , R. C. D'Souza , H. M. Palethorpe , K. A. Bradbrook , G. A. Gomez , and B. W. Day , “Personalising Glioblastoma Medicine: Explant Organoid Applications, Challenges and Future Perspectives,” Acta Neuropathologica Communications 13 (2025): 6, 10.1186/s40478-025-01928-x. 39799339 PMC11724554

[55] T. Ahmed , “Biomaterial‐Based In Vitro 3D Modeling of Glioblastoma Multiforme,” Cancer Pathogenesis and Therapy 1 (2023): 177–194, 10.1016/j.cpt.2023.01.002. 38327839 PMC10846340

[56] J. Ogawa , G. M. Pao , M. N. Shokhirev , and I. M. Verma , “Glioblastoma Model Using Human Cerebral Organoids,” Cell Reports 23 (2018): 1220–1229, 10.1016/j.celrep.2018.03.105. 29694897 PMC6892608

[57] L. Oliver , A. Álvarez‐Arenas , C. Salaud , et al., “A Simple 3D Cell Culture Method for Studying the Interactions Between Human Mesenchymal Stromal/Stem Cells and Patients Derived Glioblastoma,” Cancers 15 (2023): 1304, 10.3390/cancers15041304. 36831643 PMC9954562

[58] S. Bian , M. Repic , Z. Guo , et al., “Genetically Engineered Cerebral Organoids Model Brain Tumor Formation,” Nature Methods 15 (2018): 631–639, 10.1038/s41592-018-0070-7. 30038414 PMC6071863

[59] C. G. Hubert , M. Rivera , L. C. Spangler , et al., “A Three‐Dimensional Organoid Culture System Derived From Human Glioblastomas Recapitulates the Hypoxic Gradients and Cancer Stem Cell Heterogeneity of Tumors Found In Vivo,” Cancer Research 76 (2016): 2465–2477, 10.1158/0008-5472.CAN-15-2402. 26896279 PMC4873351

[60] M. N. Oliveira , M. M. Pillat , J. Baranova , et al., “Glioblastoma Cell Invasiveness and Epithelial‐to‐Mesenchymal Transitioning Are Modulated by Kinin Receptors,” Advances in Cancer Biology—Metastasis 4 (2022): 100045, 10.1016/j.adcanc.2022.100045.

[61] L. Garros‐Regulez , I. Garcia , E. Carrasco‐Garcia , et al., “Targeting SOX2 as a Therapeutic Strategy in Glioblastoma,” Frontiers in Oncology 6 (2016): 222, 10.3389/fonc.2016.00222. 27822457 PMC5075570

[62] S. M. Turaga and J. D. Lathia , “Adhering Towards Tumorigenicity: Altered Adhesion Mechanisms in Glioblastoma Cancer Stem Cells,” CNS Oncology 5 (2016): 251–259, 10.2217/cns-2016-0015. 27616054 PMC6040057

[63] F. Seker‐Polat , N. Pinarbasi Degirmenci , I. Solaroglu , and T. Bagci‐Onder , “Tumor Cell Infiltration Into the Brain in Glioblastoma: From Mechanisms to Clinical Perspectives,” Cancers 14 (2022): 443, 10.3390/cancers14020443. 35053605 PMC8773542

[64] I. A. W. Ho and W. S. N. Shim , “Contribution of the Microenvironmental Niche to Glioblastoma Heterogeneity,” BioMed Research International 2017 (2017): 9634172, 10.1155/2017/9634172. 28630875 PMC5467280

[65] Z. Wang , H. Zhang , S. Xu , Z. Liu , and Q. Cheng , “The Adaptive Transition of Glioblastoma Stem Cells and Its Implications on Treatments,” Signal Transduction and Targeted Therapy 6 (2021): 124, 10.1038/s41392-021-00491-w. 33753720 PMC7985200

[66] Y. Wu , B. Z. Wu , Y. Ellenbogen , et al., “Neurodevelopmental Hijacking of Oligodendrocyte Lineage Programs Drives Glioblastoma Infiltration,” Developmental Cell 60 (2025): 2420–2433.e12, 10.1016/j.devcel.2025.04.022. 40381621

[67] X. Lin , Z. Huang , and Y. Wang , “Neuronal Mechanisms Govern Glioblastoma Cell Invasion,” Neuroscience Bulletin 39 (2023): 1027–1030, 10.1007/s12264-023-01028-7. 36723779 PMC10264303

[68] S. Rosinska , G. André‐Grégoire , M. Kerhervé , et al., “Junctional Adhesion Molecule C Limits Glioblastoma Stem‐Like Cell Invasion by Regulating Integrin Adhesion at the Endothelial Interface,” Cell Reports 44 (2025): 116194, 10.1016/j.celrep.2025.116194. 40875295

[69] Y. Kim , F. S. Varn , S.‐H. Park , et al., “Perspective of Mesenchymal Transformation in Glioblastoma,” Acta Neuropathologica Communications 9 (2021): 50, 10.1186/s40478-021-01151-4. 33762019 PMC7992784

[70] C. T. Mierke , “Extracellular Matrix Cues Regulate Mechanosensing and Mechanotransduction of Cancer Cells,” Cells 13 (2024): 96, 10.3390/cells13010096. 38201302 PMC10777970

[71] A. G. Bhargav , J. S. Domino , R. Chamoun , and S. M. Thomas , “Mechanical Properties in the Glioma Microenvironment: Emerging Insights and Theranostic Opportunities,” Frontiers in Oncology 11 (2022): 805628, 10.3389/fonc.2021.805628. 35127517 PMC8813748

[72] M. Nomura , A. Spitzer , K. C. Johnson , et al., “The Multilayered Transcriptional Architecture of Glioblastoma Ecosystems,” Nature Genetics 57 (2025): 1155–1167, 10.1038/s41588-025-02167-5. 40346361 PMC12081307

[73] V. M. Ravi , P. Will , J. Kueckelhaus , et al., “Spatially Resolved Multi‐Omics Deciphers Bidirectional Tumor‐Host Interdependence in Glioblastoma,” Cancer Cell 40 (2022): 639–655.e13, 10.1016/j.ccell.2022.05.009. 35700707

[74] G. Thomas and R. Rahman , “Evolution of Preclinical Models for Glioblastoma Modelling and Drug Screening,” Current Oncology Reports 27 (2025): 601–624, 10.1007/s11912-025-01672-4. 40183896 PMC12081544

[75] H. Jo , S. Lee , M.‐H. Kim , S. Park , and S.‐Y. Lee , “Recapitulating Glioma Stem Cell Niches Using 3D Spheroid Models for Glioblastoma Research,” Biosensors 14 (2024): 539, 10.3390/bios14110539. 39589998 PMC11592235

[76] G. Safarians , A. Sohrabi , I. Solomon , et al., “Glioblastoma Spheroid Invasion Through Soft, Brain‐Like Matrices Depends on Hyaluronic Acid–CD44 Interactions,” Advanced Healthcare Materials 12 (2023): 2203143. 36694362 10.1002/adhm.202203143 PMC10238626

[77] E. Jung , M. Osswald , M. Ratliff , et al., “Tumor Cell Plasticity, Heterogeneity, and Resistance in Crucial Microenvironmental Niches in Glioma,” Nature Communications 12 (2021): 1014, 10.1038/s41467-021-21117-3. PMC7881116 33579922

[78] Y. Hu , Y. Jiang , J. Behnan , et al., “Neural Network Learning Defines Glioblastoma Features to be of Neural Crest Perivascular or Radial Glia Lineages,” Science Advances 8 (2022): abm6340, 10.1126/sciadv.abm6340. PMC9177076 35675414

[79] Z. I. Day , S. Roberts‐Thomson , Y. J. Nouri , et al., “Defining the Extracellular Matrix for Targeted Immunotherapy in Adult and Pediatric Brain Cancer,” NPJ Precision Oncology 9 (2025): 184, 10.1038/s41698-025-00956-z. 40517137 PMC12167366

[80] A. Ellert‐Miklaszewska , K. Poleszak , M. Pasierbinska , and B. Kaminska , “Integrin Signaling in Glioma Pathogenesis: From Biology to Therapy,” International Journal of Molecular Sciences 21 (2020): 888, 10.3390/ijms21030888. 32019108 PMC7037280

[81] J.‐S. So , H. Kim , and K.‐S. Han , “Mechanisms of Invasion in Glioblastoma: Extracellular Matrix, Ca^2+^ Signaling, and Glutamate,” Frontiers in Cellular Neuroscience 15 (2021): 663092, 10.3389/fncel.2021.663092. 34149360 PMC8206529

[82] R. Wei , J. Zhou , B. Bui , and X. Liu , “Glioma Actively Orchestrate a Self‐Advantageous Extracellular Matrix to Promote Recurrence and Progression,” BMC Cancer 24 (2024): 974, 10.1186/s12885-024-12751-3. 39118096 PMC11308147

[83] Q. Yu , W. Xiao , S. Sun , A. Sohrabi , J. Liang , and S. K. Seidlits , “Extracellular Matrix Proteins Confer Cell Adhesion‐Mediated Drug Resistance Through Integrin α v in Glioblastoma Cells,” Frontiers in Cell and Developmental Biology 9 (2021): 616580, 10.3389/fcell.2021.616580. 33834020 PMC8021872

[84] M. Latour , N.‐G. Her , S. Kesari , and E. Nurmemmedov , “WNT Signaling as a Therapeutic Target for Glioblastoma,” International Journal of Molecular Sciences 22 (2021): 8428, 10.3390/ijms22168428. 34445128 PMC8395085

[85] Y. Lee , J.‐K. Lee , S. H. Ahn , J. Lee , and D.‐H. Nam , “WNT Signaling in Glioblastoma and Therapeutic Opportunities,” Laboratory Investigation 96 (2016): 137–150, 10.1038/labinvest.2015.140. 26641068

[86] N. P. Ozates , B. G. Bagca , A. Asik , C. Gunduz , H. Akin , and C. B. Avci , “The Impact of Inhibiting the Hippo Signaling Pathway Effector Molecule YAP1 on In Vitro Glioblastoma and Glioblastoma Stem Cells,” Scientific Reports 16 (2026): 3679. 41577723 10.1038/s41598-025-30686-y PMC12852651

[87] G. Casati , L. Giunti , A. L. Iorio , A. Marturano , L. Galli , and I. Sardi , “Hippo Pathway in Regulating Drug Resistance of Glioblastoma,” International Journal of Molecular Sciences 22 (2021): 13431, 10.3390/ijms222413431. 34948224 PMC8705144

[88] A. Wade , A. E. Robinson , J. R. Engler , C. Petritsch , C. D. James , and J. J. Phillips , “Proteoglycans and Their Roles in Brain Cancer,” FEBS Journal 280 (2013): 2399–2417, 10.1111/febs.12109. 23281850 PMC3644380

[89] R. Azzarelli , M. Ori , A. Philpott , and B. D. Simons , “Three‐Dimensional Model of Glioblastoma by co‐Culturing Tumor Stem Cells With Human Brain Organoids,” Biology Open 10 (2021): bio056416, 10.1242/bio.056416. 33619017 PMC7928227

[90] W. Xiao , A. Sohrabi , and S. K. Seidlits , “Integrating the Glioblastoma Microenvironment Into Engineered Experimental Models,” Future Science OA 3 (2017): FSO189, 10.4155/fsoa-2016-0094. 28883992 PMC5583655

[91] W.‐Y. Park , J. M. Gray , R. J. Holewinski , et al., “Apoptosis‐Induced Nuclear Expulsion in Tumor Cells Drives S100a4‐Mediated Metastatic Outgrowth Through the RAGE Pathway,” Nature Cancer 4 (2023): 419–435, 10.1038/s43018-023-00524-z. 36973439 PMC10042736

[92] R. L. Price , J. Song , K. Bingmer , et al., “Cytomegalovirus Contributes to Glioblastoma in the Context of Tumor Suppressor Mutations,” Cancer Research 73 (2013): 3441–3450, 10.1158/0008-5472.CAN-12-3846. 23729642 PMC4136413

[93] I. Noda , “Closer Examination of Two‐Trace Two‐Dimensional (2T2D) Correlation Spectroscopy,” Journal of Molecular Structure 1213 (2020): 128194, 10.1016/j.molstruc.2020.128194.

[94] A.‐N. Cho , F. Bright , N. Morey , C. Au , L. M. Ittner , and Y. D. Ke , “Efficient Gene Expression in Human Stem Cell Derived‐Cortical Organoids Using Adeno Associated Virus,” Cells 11 (2022): 3194, 10.3390/cells11203194. 36291062 PMC9601198

[95] S. J. Lee , Z. Wu , M. Huang , et al., “Crosslinker‐Free in Situ Hydrogel Induces Self‐Aggregation of Human Dental Pulp Stem Cells With Enhanced Antibacterial Activity,” Materials Today Bio 31 (2025): 101451, 10.1016/j.mtbio.2025.101451. PMC11783010 39896283

