# Supplementary material for: Flash Assembloids: A Rapid Biofabrication of a Platform for Modeling Early Glioblastoma Invasion at the Glioblastoma–Brain Organoid Interfaces
Source: Adv Healthc Mater. 2026 May 11;15(33):e04842. doi: 10.1002/adhm.202504842 (PMC13542853; doi:10.1002/adhm.202504842)
Supplement: Supplementary file 1 [file ADHM-15-0-s001.docx]

**Flash Assembloids: A Rapid Biofabrication of Platform for Modeling Early Glioblastoma Invasion at the Glioblastoma-Brain Organoid Interfaces**

Chao Liang^a,1^, Henry Robert Howard^b,c,d,1^, Shihui Chen^b,c,e,1^, Won-Young Choi^f,1^, Summer Cao^b,c,d^, Arthur Chien^g^, Kevin Zou^b,c,d^, Michael Kassiou^h^, Fabien Delerue^i^, Ho Sang Jung^j^, Yeonju Park^k^, Young Mee Jung^k^, Yong-Dae Kwon^l^, Karrie M. Kiang^m^, Gilberto Ka-Kit Leung^m^, Ryohichi Sugimura^n^, Ann-Na Cho^b,c,d,e**^, Sang Jin Lee^a,o*^

*^a^Biofunctional Materials Division of Applied Oral Sciences and Community Dental Care,*

*Faculty of Dentistry, The University of Hong Kong, 34 Hospital Road, Hong Kong SAR, 999077, China*

*^b^School of Biomedical Engineering, Faculty of Engineering, The University of Sydney, Darlington, NSW 2008, Australia*

*^c^The University of Sydney Nano Institute (Sydney Nano), The University of Sydney, Camperdown, NSW 2006, Australia*

*^d^Brain and Mind Centre, The University of Sydney, Camperdown, NSW 2006, Australia*

*^e^Centre for Drug Discovery and Innovation, The University of Sydney, Camperdown, NSW 2006, Australia*

*^f^Department of Pathology, University of Tennessee Health Science Center, Memphis, TN, USA*

*^g^The Microscopy Unit, Faculty of Science and Engineering, Macquarie University, Sydney, NSW, Australia*

*^h^School of Chemistry, The University of Sydney, Sydney, NSW 2006, Australia*

*^i^Department of Genetics, The University of Texas MD Anderson Cancer Center, Houston, TX 77030, USA ^j^School of Biomedical Engineering, Korea University, Seoul, 02841, Republic of Korea*

*^k^Department of Chemistry, Institute for Molecular Science and Fusion Technology, and Kangwon Radiation Convergence Research Support Center, Kangwon National University, Chuncheon 24341, Republic of Korea*

*^l^Department of Oral & Maxillofacial Surgery, College of Dentistry, Kyung Hee University, Seoul 02447, Republic of Korea
^m^Department of Surgery, School of Clinical Medicine, LKS Faculty of Medicine, The University of Hong Kong, Hong Kong, Hong Kong SAR, 999077 China*

*^n^School of Biomedical Sciences, Li Ka Shing Faculty of Medicine, The University of Hong Kong, Pokfulam, Hong Kong SAR, People’s Republic of China*

*^o^College of Dentistry, Kyung Hee University, 26 Kyungheedae-ro, Dongdaemun-gu, Seoul, 02447 Republic of Korea*

^1^ These authors contributed equally to this work

^*^*^,^*^**^ These corresponding authors contributed equally to this work

Correspondence to Ann-Na Cho^**^ (Ph.D.) and Sang Jin Lee^*^ (Ph.D.)

Contact: [ann.cho@sydney.edu.au](mailto:ann.cho@sydney.edu.au) (Ann-Na Cho), [dentsj@hku.hk](mailto:dentsj@hku.hk) (Sang Jin Lee)

**Supplementary Table 1.** Antibodies used for immunostaining

| **Antibody** | **Host** | **Dilution, Catalog number, Company** |
| --- | --- | --- |
| Anti-SOX2 | Rabbit polyclonal | 1:500, #AB5603, Sigma-Aldrich |
| Anti-FOXG1 | Rabbit polyclonal | 1:250, #ab18259, Abcam |
| Anti-Laminin | Rabbit polyclonal | 1:250, #ab11575, Abcam |
| Anti-PAX6 | Rabbit polyclonal | 1:250, #901301, Biolegend |
| Anti-MAP2 | Mouse monoclonal | 1:250, #ab11267, Abcam |
| 4,6-DIAMIDINO-2-PHENYLINDOLE (DAPI) | N/A | 1:500, #D1306, Invitrogen |
| Anti-Ki67 | Rabbit polyclonal | 1:250, #ab15580, Abcam |
| Anti-Nestin | Mouse monoclonal | 1:250, #MAB5326, Sigma-Aldrich |
| Anti-Tuj1 | Mouse polyclonal | 1:500, #801213, BioLegend |
| Anti-NeuN | Mouse polyclonal | 1:500, #MAB377, Sigma-Aldrich |
| Anti-Vimentin–Alexa Fluor™ 647 | Rabbit monoclonal | 1:1500, #9856, Cell Signaling Tech. |
| Anti-EpCAM–Alexa Fluor™ 555 | Rabbit polyclonal | 1:2000, #bs-4889R-BF555, Bioss |
| Alexa Fluor™ 488 conjugated (Secondary) | Goat anti-rabbit | 1:200, #A11008, Thermo Fisher Sci. |
| Alexa Fluor™ 594 conjugated (Secondary) | Donkey anti-mouse | 1:200, #A-21203, Thermo Fisher Sci. |
| Alexa Fluor™ 647 conjugated (Secondary) | Donkey anti-mouse | 1:200, #A-31571, Thermo Fisher Sci. |

**Figure S1.** Immunocytochemistry (ICC) analysis to characterize dorsal forebrain organoids (DOs). Representative immunofluorescence images demonstrating regional identity, neuronal differentiation, and proliferative populations within human dorsal forebrain organoids as previously reported [1,2]. (a) Expression of the neuroonal progenitor marker PAX6 (magenta), confirming neuroectoderm lineage specification, with nuclei counterstained using DAPI (blue). (b) Robust neuronal differentiation indicated by TUJ1 (βIII-tubulin, red) labeling throughout the organoid structure. (c) Forebrain regional identity validated by widespread expression of FOXG1 (green). (d) Mature neuronal populations visualized by MAP2 staining (green), highlighting neurite organization and cortical-like architecture. (e) Neural progenitor and proliferative compartments identified by Nestin (red) and Ki67 (green), respectively, demonstrating the coexistence of progenitor and cycling cell populations within the developing organoid. Merged panels show combined fluorescence channels for each staining condition. Scale bars: 200 µm.

**Figure S2.** Mean displacement of GBM cells from the initial spheroid centroid over time. Distances were quantified from time-lapse phase images and converted to physical units (mm). GBM cells cultured alone (blue dot) exhibited gradual radial dispersal, whereas GBM cells in DO assembloids (red square) showed significantly greater displacement, reflecting enhanced and directionally biased invasion toward the organoid. Data represent mean ± SEM (n = 9). Linear regression analysis revealed a significantly higher rate of displacement in GBM–DO compared to GBM alone (interaction F(1,68) = 104.7, *p < 0.001).

**Figure S3.** Cellular integration and invasion in ASM model. (a) Representative H&E staining showing overall organoid morphology and preservation of ventricular-like structures within the ASM group. (b) Immunofluorescence staining for Nestin (green) and DAPI (blue) demonstrating spatially distinct cellular organization within ASM. Nestin expression delineates endogenous neural progenitor zones forming radially organized ventricular-zone-like structures with lumen organization (yellow arrows), consistent with neural rosettes intrinsic to DO development. In contrast, regions marked by loss of radial polarity, irregular tissue boundaries, and surface-associated cellular accumulation (white arrows) lack organized rosette architecture and are consistent with GBM invasion and associated disruption of host tissue structure. Insets indicated GBM adherence on DO (b-i) and invasion process (b-ii). It highlights the distinction between preserved progenitor organization and regions exhibiting tumor-associated architectural distortion. White arrow: GBM adherence and invasive location; yellow arrow: DO original structure (mesenteric)

**Figure S4.** Boxplots showing the expression levels of representative genes across ASM, DO, and GBM samples. (a) for stemness, (b) for cluster 1 (C1) of heatmap, (c) for cluster 2 (C2) of heatmap, (d) for cluster 3 of heatmap, (e) for cluster 4 of heatmap. Expression values are shown as log₂-transformed TPM. Significance annotations indicate adjusted p-values derived from DESeq2 differential expression analysis (*p < 0.05, **p < 0.01, ***p < 0.001).

**Figure S5.** Preparation of DO and GFP-labeled GBM/5OA constructs. (a) Brightfield and fluorescence imaging before implantation (-1 day) and (b) after implantation of pre-aggregated DO and GFP-labeled GBM ASM in mice subcutaneously, and an autopsy was conducted one week later. (c) Upon examination of the tissues via H&E staining, only GBM cells were present, and DOs were not visible.

**References**

1	A.-N. Cho, F. Bright, N. Morey, C. Au, L. M. Ittner, Y. D. Ke. "Efficient gene expression in human stem cell derived-cortical organoids using adeno associated virus," *Cells* 11 (2022): 3194.

2	R. Martínez-Mármol, R. Giordano-Santini, E. Kaulich, A.-N. Cho, M. Przybyla, M. A. Riyadh, et al. "SARS-CoV-2 infection and viral fusogens cause neuronal and glial fusion that compromises neuronal activity," *Science advances* 9 (2023): eadg2248.
